# Hydrophobic Core Variations Provide a Structural Framework for Tyrosine Kinase Evolution and Functional Specialization

**DOI:** 10.1371/journal.pgen.1005885

**Published:** 2016-02-29

**Authors:** Smita Mohanty, Krishnadev Oruganty, Annie Kwon, Dominic P. Byrne, Samantha Ferries, Zheng Ruan, Laura E. Hanold, Samiksha Katiyar, Eileen J. Kennedy, Patrick A. Eyers, Natarajan Kannan

**Affiliations:** 1 Department of Biochemistry & Molecular Biology, University of Georgia, Athens, Georgia, United States of America; 2 Institute of Bioinformatics, University of Georgia, Athens, Georgia, United States of America; 3 Department of Biochemistry, Institute of Integrative Biology, University of Liverpool, Liverpool, United Kingdom; 4 Department of Pharmaceutical and Biomedical Sciences, University of Georgia, Athens, Georgia, United States of America; Weizmann Institute of Science, ISRAEL

## Abstract

Protein tyrosine kinases (PTKs) are a group of closely related enzymes that have evolutionarily diverged from serine/threonine kinases (STKs) to regulate pathways associated with multi-cellularity. Evolutionary divergence of PTKs from STKs has occurred through accumulation of mutations in the active site as well as in the commonly conserved hydrophobic core. While the functional significance of active site variations is well understood, relatively little is known about how hydrophobic core variations contribute to PTK evolutionary divergence. Here, using a combination of statistical sequence comparisons, molecular dynamics simulations, mutational analysis and *in vitro* thermostability and kinase assays, we investigate the structural and functional significance of key PTK-specific variations in the kinase core. We find that the nature of residues and interactions in the hydrophobic core of PTKs is strikingly different from other protein kinases, and PTK-specific variations in the core contribute to functional divergence by altering the stability and dynamics of the kinase domain. In particular, a functionally critical STK-conserved histidine that stabilizes the regulatory spine in STKs is selectively mutated to an alanine, serine or glutamate in PTKs, and this loss-of-function mutation is accommodated, in part, through compensatory PTK-specific interactions in the core. In particular, a PTK-conserved phenylalanine in the I-helix appears to structurally and functionally compensate for the loss of STK-histidine by interacting with the regulatory spine, which has far-reaching effects on enzyme activity, inhibitor sensing, and stability. We propose that hydrophobic core variations provide a selective advantage during PTK evolution by increasing the conformational flexibility, and therefore the allosteric potential of the kinase domain. Our studies also suggest that Tyrosine Kinase Like kinases such as RAF are intermediates in PTK evolutionary divergence inasmuch as they share features of both PTKs and STKs in the core. Finally, our studies provide an evolutionary framework for identifying and characterizing disease and drug resistance mutations in the kinase core.

## Author Summary

Proteins evolve new functions through accumulation of mutations in the primary sequence. Understanding how naturally occurring mutations shape protein function can provide insights into how non-natural mutations contribute to disease. Here, we identify sequence variants associated with the functional specialization of tyrosine kinases, a large and medically important class of proteins associated with the evolution of complex multicellular functions and diseases such as cancer. We find that mutations distal from the active site contribute to functional specialization by altering the stability, activity and dynamics of the kinase core. Our findings have implications for understanding the evolution of allosteric regulation in tyrosine kinases, and in predicting the structural and functional impact of disease and drug resistance mutations in the kinase core.

## Introduction

Tyrosine phosphorylation is a fundamental mechanism by which diverse cellular processes are orchestrated and regulated in humans and other multicellular organisms. The domain that catalyzes tyrosine phosphorylation (referred to as the Protein Tyrosine Kinase (PTK) domain) belongs to a large super-family of proteins that include Serine/Threonine Kinases (STKs), Tyrosine Kinase-Like (TKL) kinases, and small molecule kinases [1–4]. PTKs form a distinct monophyletic group[4]within the protein kinase superfamily and are further sub-classified into families and sub-families based on similarities in the commonly conserved protein kinase domain [5,6]. The expansion of PTKs in multi-cellular organisms and their underrepresentation in prokaryotes and unicellular organisms had initially led to the belief that PTKs have evolved to regulate pathways associated with multi-cellularity [3,4,7,8]. However, the identification of several PTK-related sequences in unicellular protists such as *Monosiga* [9,10] have raised new questions regarding the origin, evolution, and functional niche of PTK signaling machinery [11,12]. While previous co-evolutionary analyses of PTK domains and functionally associated regulatory domains have provided important insights into the origin and evolution of phospho-tyrosine signaling machinery [3–5,7,11–16], a deeper residue-level understanding of how PTKs evolutionary diverged from other protein kinases is currently lacking. Mapping the evolutionary trajectory of the PTK domain at the residue level is necessary not only to understand their unique functions in signaling pathways, but also to understand the functional impact of numerous disease and drug resistance mutations that are found in the PTK core [5].

Some of the initial residue-level comparisons of PTK and STK sequences were originally performed in the1980’swhen fewer than 100 protein kinase sequences were available [1,17]. These initial comparisons led to the identification of key distinctive motifs in the active site of PTKs that contribute to substrate recognition and specificity. In particular, a conserved HRDL**AAR**N motif in the catalytic loop was identified as a distinctive feature of PTKs as the corresponding motif is conserved as HRDL**KPE**N in STKs. Crystal structure comparisons of Insulin receptor kinase (PTK) domain and Protein Kinase A (STK) showed that PTK-specific variations in the catalytic loop contribute to substrate recognition by creating a unique binding pocket for tyrosine residues [18–20]. In particular, the arginine residue in the AAR motif hydrogen bonds to the tyrosine hydroxyl group, and together with other PTK-conserved residues in the activation loop, contributes to substrate recognition and specificity [18,21–24].

While the structural and functional significance of active site variations in PTKs is well understood, relatively little is known about how variations distal from the active site, in particular those found in the buried core, contribute to PTK evolutionary divergence. For example, a large-scale comparison of protein kinase related sequences from diverse organisms indicated that a histidine residue (H158 in PKA) in the E-helix that is highly conserved in the hydrophobic core of diverse STKs and distantly related small molecule kinases is selectively substituted to an alanine, serine or glutamate in PTKs [25–29]. However, the structural or functional significance of this variation is not well understood. Structurally, H158 (henceforth referred to as the STK-histidine) invariantly forms a hydrogen bond with a highly conserved aspartate in the regulatory spine [30–32]in nearly all active STK crystal structures [28]. Precise positioning of the aspartate in the regulatory spine is critical for kinase activity [28,32,33], and mutation of the STK-histidine to an alanine in Aurora A kinase [33] and in PKA [34] (both STKs) completely abrogates activity, while mutation of the STK-histidine to a leucine in PKA reduces, but does not abolish, activity [34].However, the ability of PTKs to catalyze phosphoryl-transfer without the histidine raises the following key questions: How is the histidine mutation naturally accommodated in PTKs, and what, if any, is the functional significance of this PTK-specific variation?

Here we address the structural and functional significance of PTK-specific variations in the kinase core using an evolutionary-systems approach in which we quantitatively mine the massive amounts of evolutionary information encoded in protein kinase sequences from diverse organisms to formulate and test hypotheses regarding PTK evolutionary divergence. We find that the selective loss of the STK-histidine in PTKs is accompanied by the gain of PTK-specific residues in the hydrophobic core. Using Ephrin receptor kinase A3 (PTK) and Aurora A kinase (STK) as experimental model systems, we show that PTK-conserved residues in the core can at least partially structurally and functionally compensate for the loss of the STK-histidine. We propose that such compensatory evolution provides a selective advantage in PTK evolution by enabling new modes of allosteric coupling between active site and distal regulatory sites. We also identify specific TKLs as evolutionary intermediates in PTK evolutionary divergence and predict the structural and functional impact of drug resistance mutations in the PTK core.

## Results

### Evolutionary constraints distinguishing PTKs from STKs

**Fig 1:**
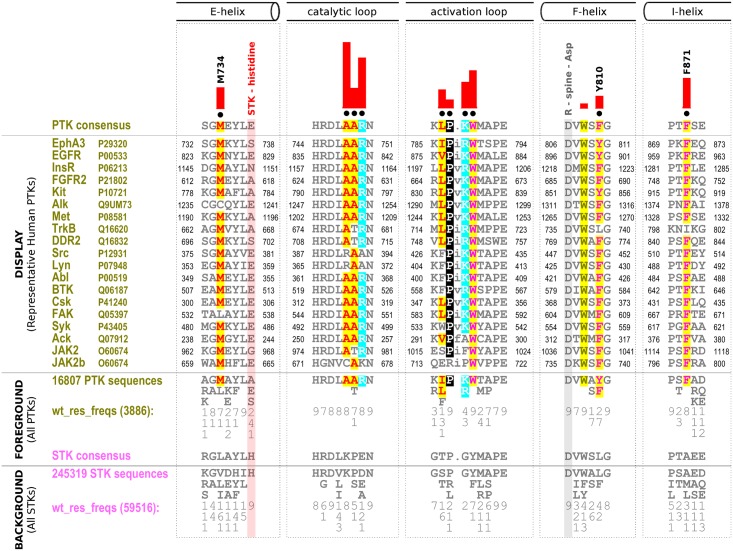
Contrast hierarchical alignment showing key evolutionary sequence constraints imposed on tyrosine kinases. An alignment of representative human PTKs from diverse PTK sub-families is shown as a display alignment. The foreground set of PTK sequences (16,807 sequences) and the background set of STK sequences (245,319 sequences) are condensed and shown indirectly via consensus patterns and by column-wise amino acid frequencies (indicated by integer tenths) observed in the entire foreground versus background alignments. For example, a ‘5’ indicates that the corresponding amino acid occurs in 50–60% of the given (weighted) sequence set. Amino acid frequencies (denoted wt_res_freq) were determined from weighted sequences to account for overrepresented kinase families and evolutionary clades in the sequence data sets, and the number in parentheses indicates the number of sequences after down-weighting for redundancy. Only divergent substrate-binding regions and key divergent positions that were further examined in this study are shown. The alignment columns that were used to partition the foreground from the background sequences by the mcBPPS procedure are marked with black dots above the display alignment, and the degree to which the foreground amino acid distribution diverges from the background amino acid distribution at each position is plotted as a red histogram. (Note that not all divergent positions are pattern positions used in mcBPPS partitioning—for example, the ‘W’ in the F-helix). Residues in the alignment are highlighted based on chemical similarity. Secondary structural elements are indicated above the alignment, and three previously unidentified PTK-conserved positions are numbered based on the human EphA3 sequence (Uniprot P29320). The regions for each PTK sequence in the display alignment are also numbered, with the corresponding Uniprot ID given next to the PTK name. The STK-histidine that is selectively lost in PTKs is highlighted in the alignment with a red rectangle. The R-spine aspartate (R-Spine-Asp) conserved in all eukaryotic protein kinases is highlighted in gray.

In order to identify residues that contribute to the evolutionary divergence of PTKs from STKs, we aligned over 250,000 protein kinase sequences from diverse organisms (see Materials and Methods) and used a Bayesian pattern partitioning procedure (see Materials and Methods for details) to identify divergent residue patterns that most distinguish PTK sequences from STK sequences. The divergent pattern corresponds to residues that are highly conserved in PTKs (also referred to as “PTK-conserved”), but strikingly different in STKs. Some of the distinguishing residues are highlighted in a contrast hierarchical alignment (Fig 1) in which representative human PTK sequences are shown as the display alignment, all PTK sequences (16,807 sequences) constitute the foreground alignment and the remaining mostly STK sequences (245,319sequences) represent the background alignment. As shown in Fig 1, in addition to residues and motifs in the catalytic loop and activation loop that have previously been noted to be important for substrate recognition[18,21–24,35], residues in the E, F, and I helices in the kinase core are highly conserved in PTK sequences but are strikingly divergent in STK sequences. For example, F871 in the I-helix is conserved as a phenylalanine in 80–90% of PTK sequences, but as an alanine, methionine, or leucine in STK sequences. The STK-histidine (S738 in EphA3) is conserved in diverse STK sequences (background alignment in Fig 1), but is substituted to a serine, alanine or glutamate in PTK sequences (foreground alignment in Fig 1). The full contrast hierarchical alignment identifying PTK-conserved residues across the whole kinase domain is shown in S1 Fig.

### Structural location and evolution of PTK-conserved residues

**Fig 2:**
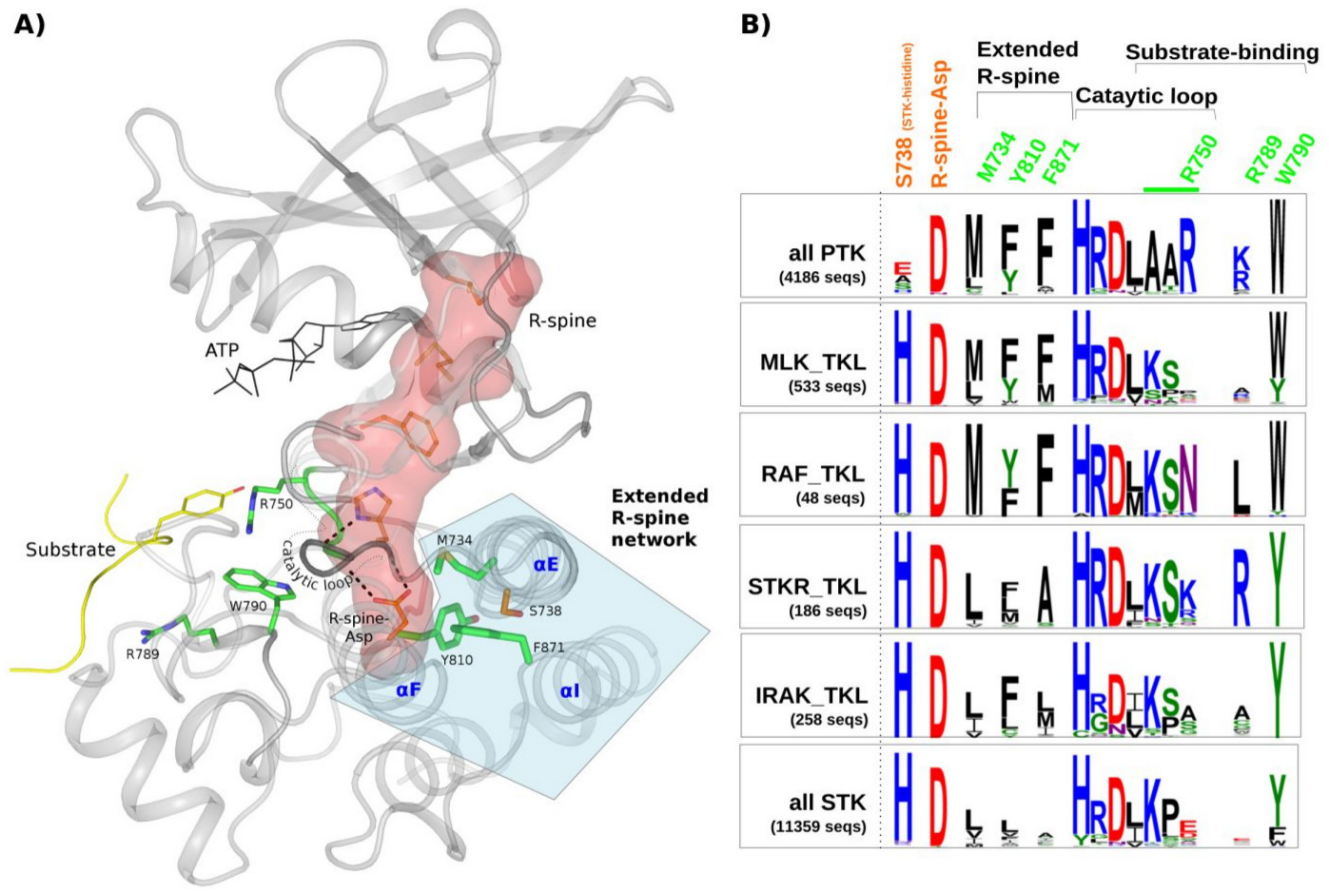
Structural location and comparisons of PTK-conserved residues in PTKs, STKs and TKLs. **A)** Key PTK-conserved residues are mapped to human EphA3 crystal structure (PDB: 3fy2) and shown in green. The extended R-spine residues are shown in stick representation and highlighted as a group in light blue. The residue at the STK-histidine position (S738) is shown in orange. The alpha helices where the substrate-binding residues are located are labeled in blue. Substrate-binding residues R750, R789, and W790 are also shown in stick representation. The substrate-binding ‘AAR’ motif of the catalytic loop is represented as the green portion of the catalytic loop, which spans the length of the small dotted lines. The R-spine is shown as a red surface representation, and key hydrogen bonds made by the F-helix-Asp and the HRD motif histidine are shown by large dotted lines. **B)** Comparative analysis of PTK-conserved patterns in PTKs, TKLs and STKs. PTK-conserved positions are indicated in green. Weblogos show the relative frequencies of residues at PTK-conserved positions in PTKs, selected TKL families, and STKs from Metazoan genomes (number of sequences indicated in the figure). The residues are numbered according to EphA3 numbering. The height of a character in each logo is proportional to its conservation in that group.

PTK-conserved residues are widely dispersed in sequence, but most spatially interact with one another to form interaction networks in three-dimensional structures. These residue networks primarily map to the C-terminal substrate-binding lobe of the kinase domain, with the exception of three residues that map to the N-terminal ATP binding lobe (S2 Fig). PTK-conserved networks in the C-lobe can be broadly classified into three regions based on their structural location: (i) a residue network (A748, A749, R750, I786, P787, R789 and W790) surrounding the substrate binding pocket (substrate-binding network in S2 Fig); (ii) a large, mostly hydrophobic network that connects the substrate binding pocket to G, F and H helices in the C-lobe (substrate-associated network in S2 Fig); and (iii) a small hydrophobic cluster (M734, Y810 and F871) surrounding the conserved aspartate in the regulatory spine [30–32](henceforth referred to as the R-spine-Asp)[31]. M734 and F871 in the cluster directly interact with the R-spine-Asp, whereas Y810 (conserved as a Leu in STKs) mediates van der Waals interactions with the side-chains of M734 and F871. Together, these three residues form an interaction network that extends the R-spine to E and I helices in the C-lobe. We refer to these PTK-conserved residues as the ‘extended R-spine-network’ (Fig 2a) and primarily focus on this network to understand the selective loss of STK-histidine in PTKs because the extended R-spine network is not only structurally proximal to the R-spine-Asp but also interacts with the R-spine-Asp in a manner analogous to the STK-histidine (see Results below). Moreover, we, and others, have recently shown that precise positioning of the R-spine-Asp is critical for kinase activation and regulation, and based on the sequence divergence described above, we hypothesized that regulation via the R-spine-Asp has diverged in unique ways in PTKs [28,32,33].

To obtain insights into how the PTK conserved extended R-spine network may have evolved during the course of evolution, we analyzed TKL families, which are believed to be evolutionary intermediates in the divergence of PTKs from STKs [2,4]. Comparisons of the patterns of conservation and variations indicate that some TKLs such as the Rapidly Accelerated Fibrosarcoma (RAF) kinase and Mixed Lineage Kinase (MLK) share features of both PTKs and STKs in the core (Fig 2b). For example, RAF and MLK share the extended R-spine network residues as well as the STK-histidine, but not the substrate-binding network residues. In particular, the substrate-binding residues, such as the AAR motif in the catalytic loop, are unique to PTKs and not observed in TKLs. Furthermore, comparisons of TKLs between diverse organisms indicates the emergence of extended R-spine network residues in early-metazoan lineages (S2 Fig). This suggests that the extended R-spine network residues evolved prior to the emergence of substrate-specificity determining features in the active site.

### Comparisons of hydrophobic core interactions in PTKs, STKs and TKLs

**Fig 3:**
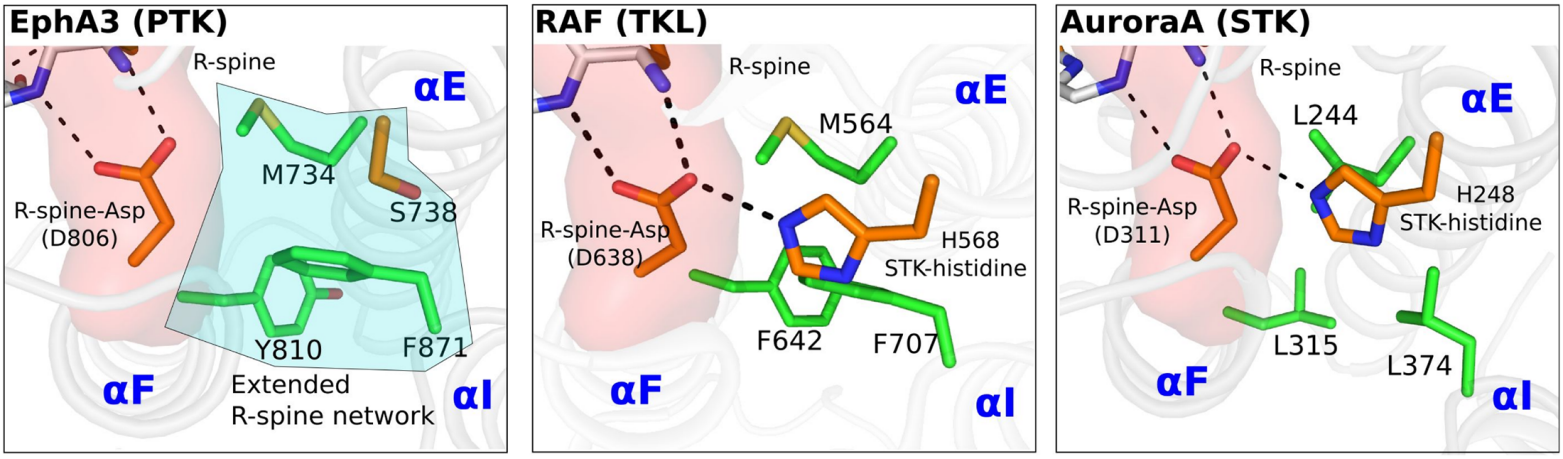
Comparative structural analysis of the extended R-spine network in EphA3, RAF and AuroraA. R-spine-Asp and STK-histidine are shown in orange and the extended R-spine network residues are shown in green. The helices where the extended R-spine residues are located are labeled in blue font. Hydrogen bonds are shown as dotted lines.

We next analyzed the structural interactions mediated by the extended R-spine network in 551 publically-available PTK crystal structures and compared the equivalent interactions in STKs and TKLs to understand the structural basis for PTK evolutionary divergence (see Materials and Methods and S3 Fig). Although we analyzed a total of 2,399 crystal structures spanning 134 families, we will use the Ephrin receptor tyrosine kinase A3 (PTK) and Aurora A kinase (STK) as models to illustrate our major findings (Fig 3) because these two kinases not only conserve the canonical PTK and STK residues, respectively, but also share a common loss-of-function phenotype when the R-spine-Asp is mutated[33,36]. As a representative example of TKLs, we use RAF kinase, which shares features of PTKs and STKs in the core (Fig 2). As observed in Fig 3, the nature of interactions associated with the R-spine-Asp is strikingly different in EphA3 (PTK), RAF (TKL) and Aurora A (STK). In Aurora A, the STK-conserved histidine forms a conserved hydrogen bond with the R-spine-Asp whereas in EphA3, the R-spine-Asp is stabilized through van der Waals interactions with F871 and M734 (Fig 3). In particular, F871 from the I-helix packs up tightly against the R-spine-Asp and energetically compensates for the loss of the STK-histidine by mediating favorable van der Waals interactions with the R-spine-Asp (S4 Fig). Notably, RAF is intermediate between STKs and PTKs in that it shares both the STK-histidine mediated hydrogen bonds as well as PTK-conserved van der Waals interactions. We also note that the I-helix is longer by one helical turn in PTKs in comparison to STKs (S5 Fig).We speculate on the functional significance of these PTK conserved features in the discussion section.

### The extended R-spine network contributes to the flexibility and stability of the catalytic core

**Fig 4:**
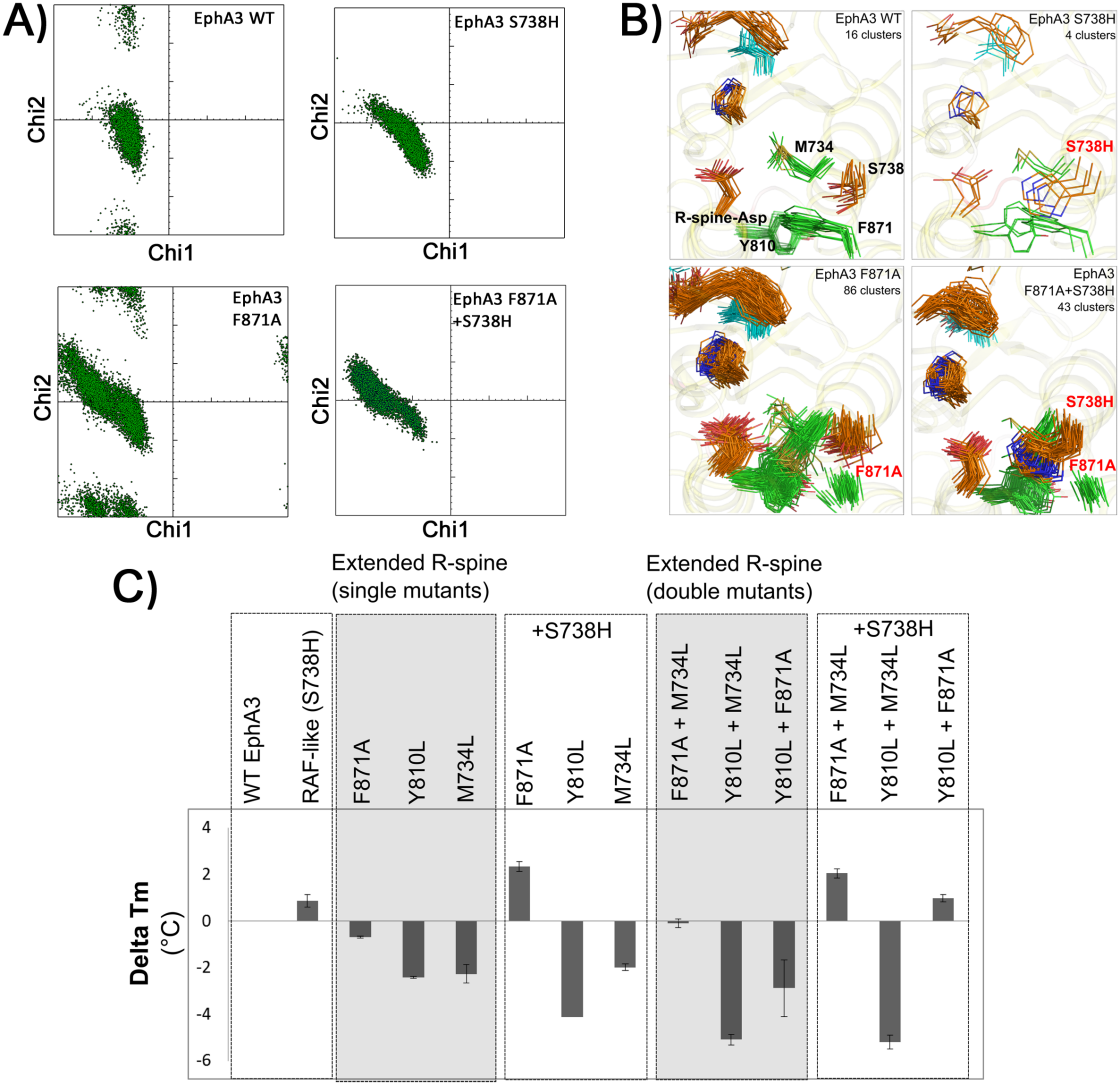
Computational and experimental stability analysis of EphA3 WT and extended R-spine mutants. **A)** Local stability analysis of EphA3 WT and mutants shown by fluctuations in side-chain conformation of R-spine-Asp. Each point corresponds to a snapshot taken at an interval of 20ps from a 200ns MD simulation trajectory. **B)** Global stability analysis of R-spine and ATP binding pocket in EphA3 WT and the extended R-spine network mutants. In each of the panels, the ATP binding site is shown in cyan, the R-spine residues are shown in orange, and the PTK-conserved residues are shown in green. For each panel, the number of clusters obtained at a clustering cutoff of 0.9 Angstroms is shown. Shown in the figure is a structural alignment of cluster centers obtained at the clustering cutoff. **C)** Thermal stability change of EphA3 and mutants are shown as histograms. Changes in Tm were calculated by subtracting Tm value of WT from Tm value of mutant. The mutants are grouped by single and double mutants, and single and double mutants with the addition of STK-histidine are shown as +S738H). The Tm values were calculated from triplicates, with standard deviations indicated above the histogram.

Because the R-spine is a flexible structural motif that is dynamically assembled upon kinase activation, we hypothesized that the extended R-spine network contributes to dynamic allostery by altering the stability and flexibility of the kinase core. To test this hypothesis, we performed molecular dynamics (MD) simulations on WT and mutant EphA3, where we substituted PTK-conserved residues in the extended R-spine network with those observed in STKs and monitored the conformational flexibility of the R-spine-Asp during the course of the simulation. We also generated a RAF-like state in EphA3 by substituting S738 to a histidine (S738H). Notably, when the extended R-spine network residues are substituted, the conformational freedom of the R-spine-Asp increases in comparison to WT EphA3 (Fig 4a and S6 Fig). In particular, substitution of F871 to an alanine (F871A in Fig 4a) or replacing all three residues in EphA3 by those observed in Aurora A (F871L+M34L+Y810L triple mutant)(S7 Fig) increases the conformational freedom of the R-spine-Asp. The conformational freedom of the R-spine-Asp, however, is reduced and is closer to WT levels when the STK-histidine is added to the F871A background (F871A+S738H in Fig 4a) or the triple mutant background (S7 Fig). Interestingly, a RAF-like state in EphA3 (S738H in Fig 4) also reduces conformational freedom of the R-spine-Asp relative to WT. Also, the conformational freedom of R-spine-Asp in WT EphA3 is higher relative to AuroraA, and this trend is broadly observed when the R-spine chi-chi2 angle distributions are compared across a diverse panel of PTKs and STKs (S6 Fig).

In addition to local dynamics, the extended R-spine network substitutions alter the global dynamics of the kinase domain by increasing fluctuations of the R-spine residues and the ATP binding pocket. As shown in Fig 4b, the number of distinct conformational states identified by clustering the molecular dynamics trajectories (see Materials and Methods) is significantly higher (86 clusters) for the F871A mutant relative to WT EphA3 (16 clusters). The M734L (38 clusters) and Y810L (39 clusters) mutants also show increased fluctuations in the R-spine and ATP binding pocket when compared to WT (S8 Fig). In the case of F871A (86 clusters), both local and global fluctuations are reduced when the STK-histidine is introduced in the F871A background (43 clusters for F871A+S738H, see Fig 4b). Thus, the STK-histidine appears to partially compensate for the destabilization caused by the extended R-spine mutants.

The trends observed in MD simulations are also corroborated by experimentally determined thermal stability measurements of WT and mutant EphA3 proteins using Differential Scanning Fluorimetry (DSF) (Fig 4c and S9 Fig). These measurements indicate that the RAF-like state (S738H mutant) is more stable than WT EphA3. In contrast, mutations in the extended R-spine network are destabilizing and the destabilizing effect of some of the mutations, such as F871A, can be rescued through the addition of the STK-histidine (+S738H) (Fig 4c). Consistently, the stability of double mutants containing F871A (F871A+Y810L and M734L+F871A) can also be rescued by STK-histidine. However, addition of the STK-histidine in M734L and Y810L single and double mutant backgrounds does not restore stability. In a reciprocal experiment, destabilization of Aurora A by mutating the STK-histidine (H248A) was partially restored by introducing the extended R-spine network residues (L244M introduces a residue equivalent to M734, and L374F introduces a residue equivalent to F871) (S9 Fig). Our thermostability analysis provides compelling evidence that F871 in the extended R-spine network plays a dominant role in contributing to both the dynamics and stability of the PTK catalytic core, and likely compensates structurally for the absence of the STK-histidine residue in the tyrosine kinase EphA3. To evaluate extended R-spine network components further, we assessed the activity of EphA3 mutants from a variety of sources, using a combination of enzyme and autophosphorylation assays.

### Impact of extended R-spine network mutants on EphA3 catalytic activity

**Fig 5:**
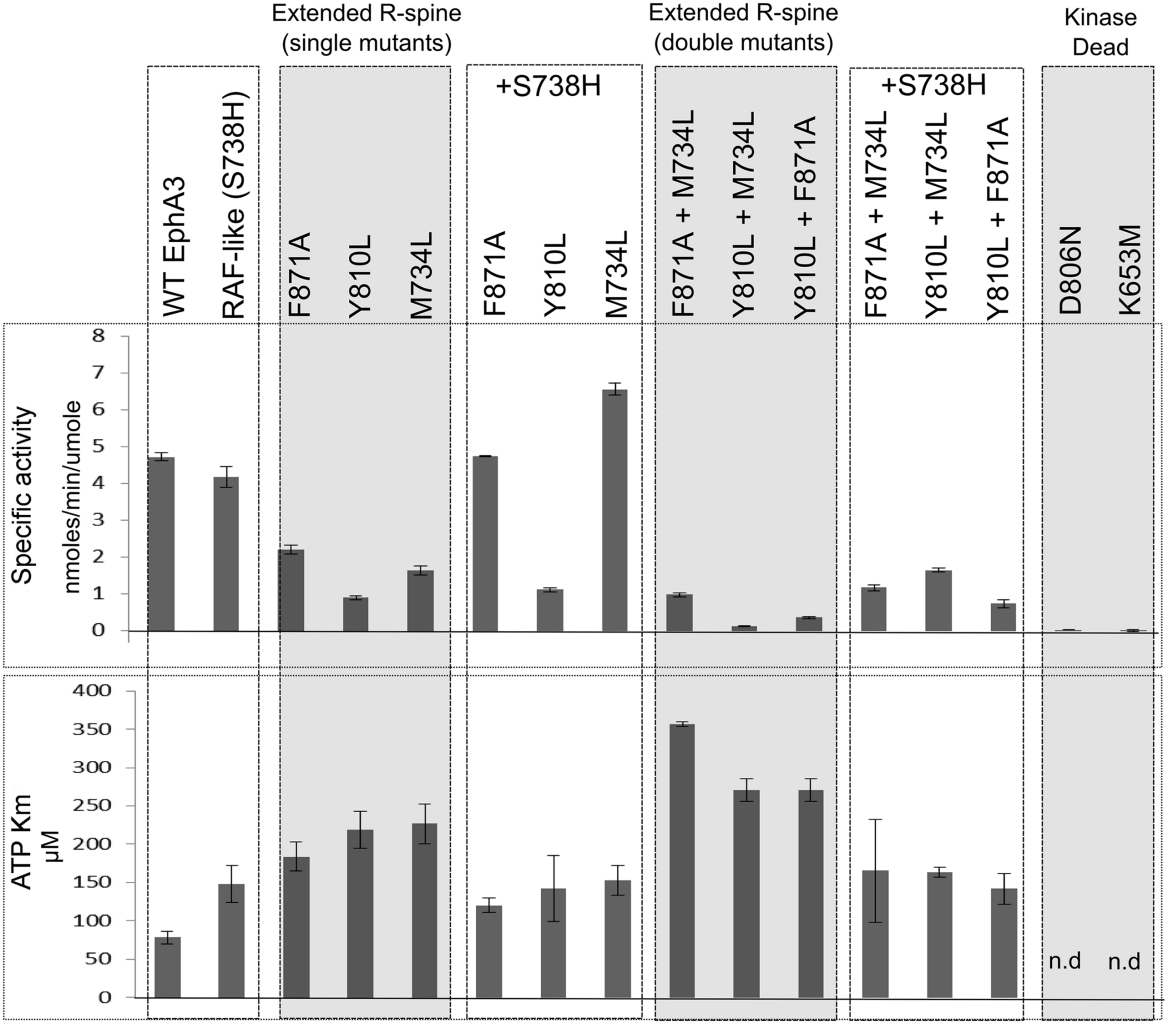
Specific activity and ATP Km of EphA3 WT and extended R-spine network mutants. The single extended R-spine mutants exhibit loss of specific activity and an increase in ATP Km that can be partially rescued by addition of STK-histidine. Extended R-spine double mutants show partial restoration of specific activity with the addition of STK-histidine, but not to WT levels. Partial rescue of ATP Km is also seen for double mutants. No activity was observed for the kinase dead control (K653M) or for the R-spine-Asp mutant (D806N). For each mutant, ATP Km was measured at 12 ATP concentrations and replicated at least 5 times for each mutant. The histogram and error bars represent the average and standard deviation of values from replicate experiments.

To evaluate whether the extended R-spine network impacts kinase catalytic functions, we first measured Km values for ATP and a peptide substrate for WT and mutant EphA3 using an NADH coupled assay (see Materials and Methods). As a control, we assayed for the R-spine-Asp mutant (D806N), which was previously shown to abolish catalytic activity in other kinases [33,34,36]. As expected, the D806N mutation completely abolishes EphA3 activity, as does the kinase-dead control K653M, in which the ATP coordinating β3 lysine is substituted by a methionine (Fig 5 and S1 Table). In contrast, the extended R-spine network mutants reduce activity to various levels as compared to WT, but do not abolish activity (Fig 5). The reduction in activity is largely due to impaired ATP binding/ADP release, as indicated by higher ATP Km values for mutants relative to the WT, with little change in peptide Km values (Fig 5 and S1 Table). Furthermore, the double mutants (M734L+F871A, Y810L+F871A and M734L+Y810L) increased ATP Km and decreased specific activity by several fold in comparison to single mutants and WT.

To test whether the reduced activity of the extended R-spine network mutants can be rescued by the STK-histidine, as suggested by MD studies and thermal stability assays, we measured the activity of extended R-spine network mutants with the addition of STK-histidine (+S738H). Notably, addition of the STK-histidine rescued the impaired activity of the extended R-spine network mutants to varying degrees (Fig 5).In particular, the rescue of F871A and M734L was more pronounced in comparison to Y810L. Rescue of double mutant activity, however, was not as pronounced as the single mutants. It should be noted that the RAF-like intermediate (S738H mutant) itself increases ATP Km relative to WT, even though the specific activity of S738H is comparable to the WT. Additional mutations such as M734V and Y810V with and without STK-histidine were also explored to determine the effects of cavity size on activity. As shown in S2 Fig, mutations that are predicted to increase cavity size also increase ATP Km and addition of STK-histidine in the M734V and Y810V mutant background reduces ATP Km.

We next replaced residues in Aurora A kinase with those observed in PTKs to further test the hypothesis that the extended R-spine network functionally mimics the STK-histidine. We found that the addition of extended R-spine network residues increased Aurora A activity relative to the inactive mutant lacking the STK-histidine (H248A) (S11 Fig). Taken together, these data support the hypothesis that the extended R-spine network residues, in particular F871, mimics the functions of the STK-conserved histidine (H248).

**Fig 6:**
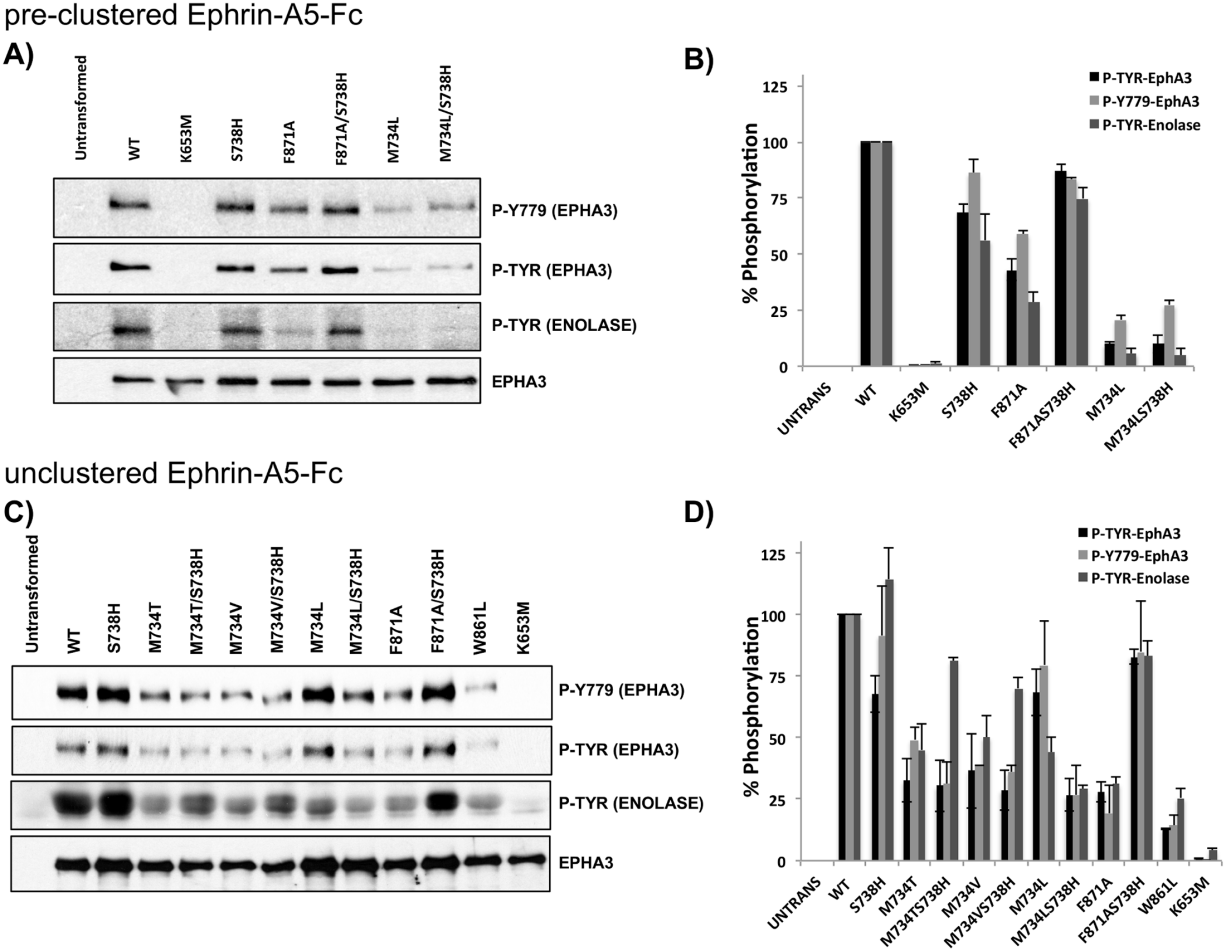
*In vitro* kinase assays probing for EphA3 autophosphorylation and enolase phosphorylation using full length EphA3 constructs in HEK293T cells. **A)** HEK293T cells stimulated with pre-clustered Ephrin-A5-Fc. The EphA3 K653M mutant was used as a kinase-dead control. Phosphorylation of WT and mutant EphA3 was detected using phospho-tyrosine (phospho-EphA3 and phospho-enolase) and EphA3 activation loop (Y779) antibodies. **B)** Quantification of Western blot shown in (A). The histograms represent average values from 3 replicate experiments, with standard deviations shown as error bars. Values were normalized using WT as reference. **C)** Phosphorylation in un-clustered HEK293T cells. Phosphorylation of WT and mutant EphA3 was probed as described in A. **D)** Quantification of Western blots presented in (C). The histograms represent average values from 3 replicate experiments, with standard deviations shown as error bars. Values were normalized using WT as reference.

To determine whether the effects of the extended R-spine network mutants also impact phosphorylation of protein substrates, we next examined the auto-phosphorylation of full-length EphA3 in HEK293T cells incubated with the Ephrin A3 ligand Ephrin-A5. Strengths of this assay are that Ephrin-A5 represents a physiological ligand that induces Ephrin A3 activation, and that the output from Ephrin-A5 can be modulated through antibody-mediated clustering [37–39]. For auto-phosphorylation and substrate phosphorylation, we specifically focused on F871A and M734Lsubstitutions, because addition of STK-histidine in these two mutant background significantly altered specific activity in the NADH coupled assay. For our auto-phosphorylation assays, we first exposed cells to clustered Ephrin-A5-Fc ligands, which robustly activate EphA3 activity (Fig 6A and 6B). We also performed our assays in the absence of pre-clustering (Fig 6C and 6D). In parallel, we probed for phosphorylation of enolase, a generic tyrosine kinase substrate (see Materials and Methods for details). Consistent with the NADH coupled assay, F871Asubstitution reduced EphA3 autophosphorylation and substrate phosphorylation using both the pre-clustered and unclustered stimuli, and this loss of function phenotype could be rescued to near WT activity levels by the addition of the STK-histidine (F871A/S738H in Fig 6). In contrast, addition of STK-histidine in the M734L background displayed different phenotypes depending on the activation state of EphA3. In particular, in the pre-clustered form, activity of the double mutant, M734L/S738H, was comparable to M734L, whereas in the unclustered form auto-phosphorylation and substrate phosphorylation of M734L/S738H was reduced compared to M734L.

### The extended R-spine network mutants alter sensitivity and selectivity of kinase inhibitors

**Fig 7:**
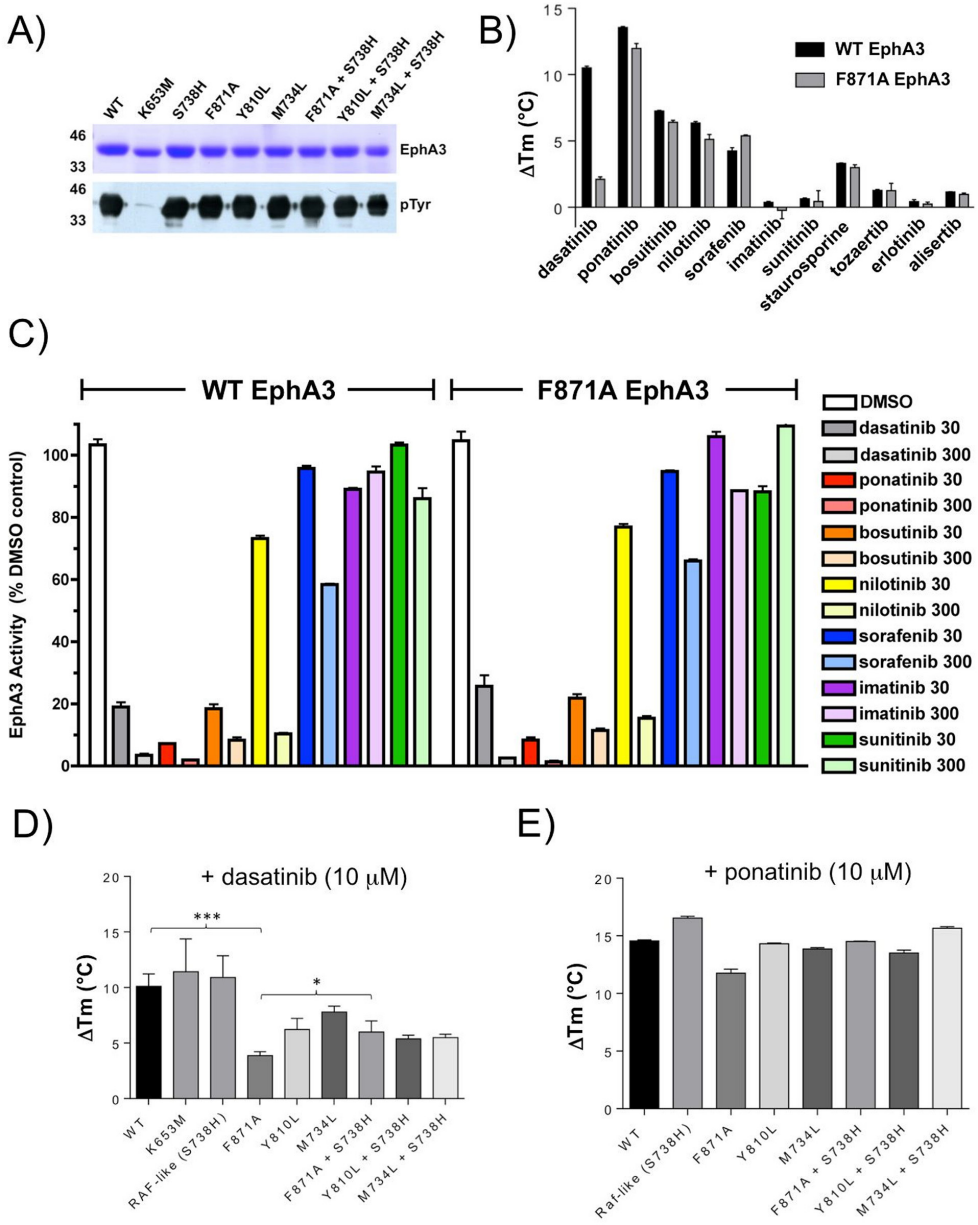
Comparative DSF and peptide-based kinase assay of EphA3 extended R-spine mutants in the presence of various tyrosine kinase inhibitors. **A)** Analysis of EphA3 WT and extended R-spine mutant proteins. 5 μg or 1 μg of the indicated purified EphA3 proteins were stained with Commassie Blue (top panel) or immunoblotted with a phosphotyrosine antibody (4G10, bottom panel). Increased electrophoretic mobility and lack of autophosphorylated tyrosine are evident in kinase dead (K653M) EphA3 when compared to the other EphA3 proteins analysed. **B)** Changes in thermal stability in WT and F871A EphA3 measured upon 10μM inhibitor binding. Of the panel of 11 type I and type II kinase inhibitors tested, all induce similar effects on thermostability in both WT EphA3 and the F871A mutant, with the notable exception of dasatinib. WT EphA3 is stabilized to a much greater extent in the presence of dasatinib than the F871A mutant.**C)** Effects of inhibitors at the indicated concentrations (nM) on WT EphA3 and F871A-catalyzed peptide tyrosine phosphorylation. Activity is normalized to the respective DMSO control with no added inhibitors. The efficacy of inhibition by various inhibitors directly mirrors trends in the thermostability changes induced by inhibitor binding observed in B). **D, E)** Thermostability effects of dasatinb (D) and ponatinib (E) on full panel of extended R-spine EphA3 mutants. The stabilization effects exhibited by dasatinib differ to those for ponatinib, and this trend is observed for all extended R-spine mutants. Mean ΔTm values ± SD from duplicate experiments were calculated by subtracting the control Tm value (DMSO, no inhibitor (S12 Fig)) from the Tm value measured in the presence of inhibitor. * p value <0.05, *** p value <0.001 (Student’s T test).

We next tested potential effects of the extended R-spine network on the binding and selectivity of a panel of clinically-relevant kinase inhibitors using DSF and peptide-based kinase assays. DSF has the advantage of reporting thermal stability changes induced by inhibitor binding, which in turn is dependent on specific EphA3 conformations present in solution, whilst kinase assays permit quantification of enzyme activities. The broad differentiation of most kinase inhibitors into type I (binding preference for active kinase conformers) and type II (binding preference to inactive kinase conformers) makes this combination approach potentially useful for probing the structural and conformational features of kinases and their mutants. Previous studies employing kinase assays demonstrated a broad susceptibility of EphA3 to inhibition by various tyrosine kinase inhibitors, several of which were assayed in this study [40,41]. As shown in Fig 7A, bacterially-expressed truncated EphA3 WT and extended R-spine mutants proteins were purified to similar levels and with the exception of kinase-dead EphA3 (K653M), which also migrated slightly more quickly than all other EphA3 proteins, exhibited similar levels of tyrosine autophosphorylation when assessed by western blot. Subsequently, the thermal stability of this mutant panel (Tm values) was re-assessed using a highly sensitive DSF protocol, demonstrating a similar change in stability profiles for mutants in the presence of 4% (v/v) DMSO solvent when compared to our earlier stability assays, where a ΔTm value was calculated relative to WT EphA3 (compare S12 Fig and Fig 4c). Next, we more closely investigated the F871A mutant, whose dynamics and stability are markedly different to WT EphA3. Interestingly, both WT and F871A EphA3 exhibited strong compound-induced stabilization in the presence of a panel of clinical PTK inhibitors, including dasatinib, ponatinib, bosutinib, nilotinib and sorafenib, but were much less responsive to imatinib, sunitinib and erlotinib, and to the Aurora A inhibitors tozaertib (VX-680) [42] and alisertib (MLN8237) [43]. Of note, the F871A mutant exhibited essentially no stabilization in the presence of the type I pan-kinase inhibitor dasatinib [44], although it showed a similar degree of stabilization to WT with all other compounds tested, including the type II pan-kinase inhibitor ponatinib [41](Fig 7b). To validate the inhibitor-induced thermal melt assay, we next assessed effects of PTK inhibitors using a kinase assay that directly monitors EphA3 enzymatic peptide phosphorylation in real time (see Materials and Methods). As shown in Fig 7c, the compounds inhibited EphA3 WT and F871A catalytic activity in the same rank order of potency identified by DSF. Interestingly, the IC_50_ values for the two most potent inhibitors (ponatinib and dasatinib) were essentially identical for both WT and F871A proteins (S12 Fig). Finally, the absence in thermal stabilization observed in F871A in the presence of dasatinib was recapitulated across the R-spine mutant panel (Fig 7d), and the introduction of S738H in the F871A background partially rescued this effect, consistent with a key allosteric role for F871 in coupling the inhibitor binding (ATP) site and the R-spine network. In contrast, the highly conserved stabilizing effect of ponatinib across the panel of EphA3 mutants suggests decreased requirements in the network for stabilization evoked by this ‘type II’ kinase inhibitor (Fig 7e).

## Discussion

**Fig 8:**
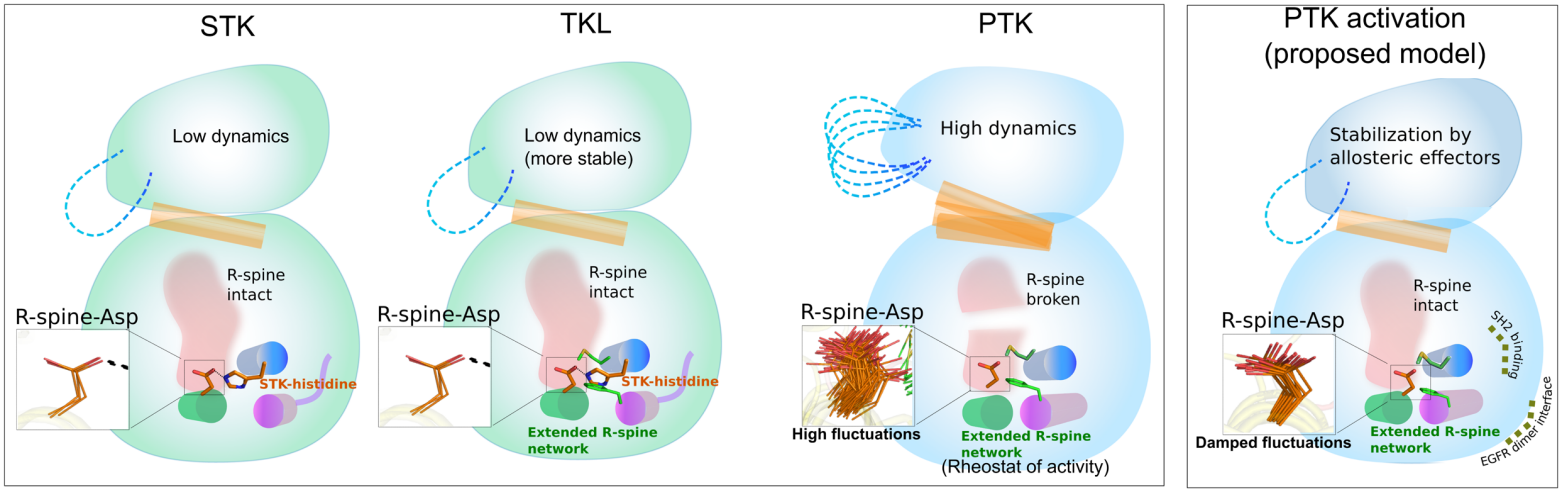
A schematic showing the evolution of PTKs from STKs with a TKL intermediate. Both STKs and TKLs conserve the STK-histidine that increases stability and reduces dynamics of the R-spine. The emergence of a more stable TKL intermediate due to conservation of both extended R-spine network and STK-histidine facilitated the loss of STK-histidine in PTKs, resulting in increased conformational entropy of the kinase domain. Shown in the schematic are C-helix (orange cartoon), the R-spine (light red blob), and the E, F and I helices that house the extended R-spine network residues. Increased dynamics may occur due to increased thermal fluctuations of the R-spine-Asp (shown in inset in stick representation). These increased dynamics may be quenched by various activating mechanisms such as receptor dimerization, regulatory domain binding (i.e. SH2 and SH3) and allosteric modulators binding to the myristic acid binding site in Abl tyrosine kinase.

PTKs have evolutionarily diverged from STKs through the accumulation of mutations in the shared catalytic domain. Here, we have investigated how mutations in the hydrophobic core of PTKs contribute to evolutionary divergence, using the intracellular TK domain of EphA3 as an experimental model. We have specifically characterized a network of residues that both associate with, and extend the regulatory spine to distal regions of the kinase domain that are currently very poorly studied. Our data suggest that the extended R-spine network, in particular a highly conserved phenylalanine residue (F871) in theEphA3 I-helix, structurally and functionally mimics the functions of an STK-conserved histidine, which has been selectively lost in PTKs. Importantly, we show that this core variation can alter the dynamics, stability, activity and inhibitor sensing by the EphA3 kinase domain, making it a useful new model for evaluation of regulatory amino acid networks amongst PTKs. Why would altered stability and dynamics of the core be important for PTK functions and how can it provide a selective advantage during PTK evolution? One possibility is that partial destabilization of the core by hydrophobic core mutations increases the regulatory potential of the kinase domain by enabling new modes of stabilization and regulation through interactions with auxiliary domains and small molecule effectors. Consistent with this view, Abl and Src tyrosine kinases, are stabilized when the regulatory SH2 domain binds to the longer I-helix [45,46], which according to our structural comparisons is uniquely found amongst PTKs. This may explain why the PTK-conserved F871 is located in the I-helix. Likewise, the allosteric regulation of Abl kinase [47] by myristic acid [45]and GNF-2/5 inhibitors [48]may involve the extended R-spine network, since the myristoyl group and the inhibitor bind to a hydrophobic groove formed between E, F, H and I helices in the catalytic core (Fig 8) [49]. Thus, the newly characterized extended R-spine network may function as a dynamic “sector” [50,51] to modulate activity and function in diverse ways in the PTKs (Fig 8).

A regulatory role for the extended R-spine network is also supported by our enzyme and DSF studies, which show that mutations in the extended R-spine network not only affect catalytic mechanisms (as shown by increased Km values for ATP) but also affect sensing of structural differences induced by a small molecule inhibitor that binds in the ATP site, leading to changes in thermal stability. The latter effect is evidenced by unfolding differences in the F871A EphA3 mutant in the presence of dasatinib, an inhibitor known to preferentially target the active conformation of tyrosine kinases [44]. In particular, the strong correlation between effects of extended R-spine network mutations on kinase activity (Figs 5 and 6) and a disconnect between dasatinib inhibition and dasatinib stabilization effects (Fig 7) argue that communication between an occupied ATP-binding site and the extended R-spine network exists. A second possible interpretation of these data are that the majority of F871A EphA3 protein resides in an (inactive) conformation with very low affinity for the type I inhibitor dasatinib (which generally targets ‘active’ kinase conformations), but essentially identical affinity for ponatinib (which usually targets ‘inactive’ kinase conformation). Indeed, it was recently shown that the presence of certain amino acids at extended R-spine equivalent positions in other kinases likely correlates with their ability to bind to Type II inhibitors [52]. We believe that careful structural validation will be needed to explain these phenomena more clearly. Likewise, the complex kinetic properties exhibited by a RAF-like state in EphA3 needs to be further characterized using detailed transient kinetics approaches.

A detailed mechanistic understanding of the PTK-specific loss of the STK-histidine is now considered to be essential, since many currently known and approved PTK-targeting inhibitors bind to conformations that are thought to be intermediates in ADP release steps, such as the ‘DFG-out’ conformation [28,53]. Here, we propose that the extended R-spine network can potentially contribute to regulation of catalysis by mimicking the functions of the STK-histidine. In particular, F871 in the I-helix appears to best mimic the functions of the STK-histidine, since addition of the STK-histidine in the F871A mutant background rescues catalytic activity both in EphA3 kinase domain construct as well as in a full length EphA3protein.In contrast, addition of the STK-histidine to the M734L mutant background only partially restores activity in the kinase domain construct (Fig 5), but not in the full length protein, suggesting that regions outside of the kinase domain (*e*.*g*. the C-terminal SAM domain and the extracellular ligand binding domain)probably also contribute to the functions of the extended R-spine network. The M734L mutant is clearly more sensitive (less active than WT) to the use of clustered Ephrin-A5-Fc ligand, and we note that this ligand is associated with the stimulation of robust signaling [39]. Although we do not currently have a clear explanation as to why M734L substitution is selectively altered byEphA5 ligand clustering, one possibility is that pre-clustering of ligands alters the rate of either M734L auto-phosphorylation or dephosphorylation. Also, differential stability of the phosphorylated Y779 residue on the M734L mutant might be important during extraction, potentially as a function of the activity of tyrosine phosphatases, whose total activity might be altered in clustered vs. unclustered lysates. This finding lead us to suggest that additional factors such as spatial and temporal aspects of EphA3 signaling need to be considered in order to fully understand the role of the extended R-spine network.

In conclusion, our studies support a step-wise model of PTK evolutionary divergence in which emergence of a RAF-like intermediate facilitates PTK diversification and neo-functionalization. Mutation of the STK-histidine is deleterious in contemporary STK sequences, however, in the presence of the extended R-spine network, mutations at the STK-histidine position can be tolerated, as indicated by the increased stability of RAF-Like state in EphA3. Thus, the presence of both the STK-histidine and the extended R-spine network in TKLs (such as RAF) reflects functional redundancy that acts to facilitate evolution of PTKs. Such evolution of new functions by selective loss of redundant residue networks has been observed in other proteins and is considered a general aspect of protein functional divergence [54–59]. It should, however, be mentioned that our proposed model of PTK evolutionary divergence does not consider cases of convergent evolution such as bacterial tyrosine kinases (BY-kinases), which are structurally and mechanistically distinct from PTKs [60–62].

The proposed model of PTK functional specialization has implications for understanding the impact of disease-linked mutations in the protein core. For example, the M734T EphA3 mutation (S1 Table) mimics a frequently occurring drug resistance mutation in Abl tyrosine kinase (M351T in Abl) [63,64], which alters a PTK-conserved residue in the extended R-spine network. The mechanism by which the M734T equivalent mutation contributes to drug resistance in tyrosine kinases is not apparent from crystal structure analysis because the mutation is distal from the ATP/drug binding site. However, based on our functional characterization of the extended R-spine network in EphA3, we predict that the M734T mutation may induce drug resistance by increasing the conformational flexibility of the R-spine-Asp in Abl. Consistent with this prediction, the M734T mutation in EphA3 increases the Km value for ATP by nearly four-fold (S1 Table), and reduces EphA3 auto-phosphorylation and substrate phosphorylation relative to WT (Fig 6C). It will be interesting to assess the effects of mutations at this site on the broad spectrum of EphA3 inhibitors identified in this study using two complementary assays (Fig 7). The role of protein dynamics in drug binding has been illustrated in recent work on the evolution of Src and Abl kinases [65,66] and explains why the difference in affinity for imatinib and other drugs may be as high as 100-fold despite very high sequence and structural similarity at the drug binding site (*e*.*g*. only four conservative substitutions exist between Src and Abl). Our validation that EphA3 is a potent target for the closely-related nilotininb and ponatinib, yet is essentially untouched by imatinib (Fig 7) make this tyrosine kinase a new model for further studies in this area, particularly with respect to the extended R-spine network. Indeed, NMR and fast kinetic methods also previously concluded that distal site dynamics affect induced-fit mechanisms and give rise to different affinities for a given compound [65]. Based on our studies, we propose that sub-family-specific variations in the core, influenced by additional epistatic networks, may contribute to variable ATP and drug binding affinities by altering core dynamics, perhaps specifying conformers with some preference between type I and type II kinase inhibitors. Finally, and more generally, hydrophobic residue networks therefore appear to be an important and under-appreciated protein characteristic, which are important for multiple functions in addition to protein folding and stability. Close studies of such networks in PTKs, especially those surrounding substrate-binding regions, will be an important next step in characterizing the PTK regulatory machinery and its exploitation for the molecular design, prediction and evaluation of small molecule interactions.

## Materials and Methods

### Identification of PTK specific sequence patterns and phylogenetic analysis

We used a dataset of 262,126 eukaryotic protein kinase sequences identified using previously curated kinase sequence profiles [67]. The 262,126 sequences were aligned using MAPGAPS[68], a rapid and accurate alignment procedure. This was used as an input for mcBPPS [69], a Bayesian pattern based partitioning algorithm, which identifies residue positions in the alignment that most distinguish PTK sequences (‘foreground’) from STK (‘background’ sequences). In our analysis, we excluded tyrosine kinase-like sequences (TKLs), which share some of the PTK features in the kinase core.

Uniprot database was used to map each of the 262,126 protein kinases to their corresponding NCBI taxonomic ids [70], and the mapped taxonomy was used for phylogenetic analysis and WebLogo display [71].

### Structural analysis and pairwise residue energy analysis

Crystal structural analysis was carried out on 4100 protein kinase chains obtained from PDB. Sequences corresponding to the kinase domain were aligned using MAPGAPS to map structurally equivalent positions. The aligned positions were manually evaluated for accuracy and pair-wise interaction energy between residues was calculated using AMBER force field[72].

### Molecular dynamics simulation

Molecular dynamics was performed using Gromacs version 4.6.4. Coordinates from PDB 3fy2 (for EphA3) and PDB 1mq4 (Aurora A) were used as starting structures. Missing residues in these structures were modeled using Modloop [73]. All heteroatoms and water atoms from the PDB structures were removed, and phospho amino acids were replaced by the unphosphorylated forms for simulation. Proteins were placed in a dodecahedron periodic box with each side at least 1nm away from the protein. The proteins were parameterized using AMBER99SB-ILDN force field and TIP3P water model was used. Energy minimization was done using steepest descent and conjugate gradient methods after applying position restraints to heavy atoms. Long range electrostatics was used with PME method with a cutoff of 10 nm. In order to accelerate the simulations, virtual site construction for all hydrogens was carried out and a time step of 5 fs was used for the simulations. Temperature equilibration was done in an NVT ensemble for 500 pico seconds at a temperature of 35 C with a Berendsen thermostat. Pressure and density equilibration was done for 1 nano second using Berendsen thermostat and Berendson barostat. For production runs, Parinello-Rahman barostat and Nose-Hoover thermostat were used. Production runs were for 200ns with frames written every 2 pico seconds. Cluster analysis was carried out using g_cluster module of Gromacs.

### Expression and purification of EphA3 and Aurora A wild type and mutant proteins

The wild type human EphA3 was obtained from Addgene (plasmid id 25136). EphA3 plasmid comprises the kinase domain and the juxtamembrane region with an N-terminal 6X His tag in pET28a LIC vector under a T7 promoter. Mutagenesis was carried out using high fidelity Phusion DNA polymerase (from NEB). The plasmids were expressed in Rosetta II BL21 cells; overnight induction was carried out at an O.D 600 of 1.0 with 0.5 mM IPTG at 15°C. Cells were harvested after growing overnight and re-suspended in Lysis buffer (50mM phosphate at pH 8.0, 0.5M NaCl and 1mM PMSF) followed by sonication. The cell debris was discarded and the supernatant was applied on Ni-NTA agarose column and purification was done using a batch purification protocol, with additional gel filtration and MonoQ chromatography where appropriate. The concentration of protein purified was estimated by SDS PAGE or Coomassie dye-binding (Fig 7A), employing 1mg/ml BSA as a standard. Aurora A construct creation, expression and purification was performed as described previously [33].

### Kinase activity assays using ATP/NADH coupled assay or direct peptide phosphorylation

Kinase activity was measured using a PK/LDH coupled assay (see S1 Method for details). Briefly, the mutants were purified and incubated with 10mM ATP and 20 mM MgCl_2_ to allow for complete autophosphorylation. EphA3 WT and mutants were added to reaction buffer consisting of 100mM Tris (pH 8.1), 300mM NaCl, 20mM MgCl_2_, 1mM PEP, 500μM NADH and peptide substrate (NH2-KQWDNYEFIW-COOH)[74]. Reactions were followed for 1 hour at an interval of 1.5 minutes, initial velocities were calculated from time course data, and the initial velocities were used to fit to a pseudo first order Michaelis-Menten equation (curve fits in S10 Fig). ATP and peptide Km was determined at saturating ATP and peptide concentrations, respectively. For direct phosphorylation assays, 100 ng of WT or F871A EphA3 was evaluated with 5 μM EphA3 fluorescent peptide substrate (5-FAM-EFPIYDFLPAKKK-CONH_2_). Phosphorylation was limited to <20% in the presence of 0.2 mM ATP, 5 mM MgCl_2_, and inhibitor (final DMSO concentration 1% (v/v)). Peptide phosphorylation was calculated directly by differentiating phosphopeptide:peptide ratios. Further details are provided in the Supplementary methods (S1 Method).

### Differential Scanning Fluorimetry (DSF) for assessing global thermostability of wild type and mutant proteins

Thermal melt assays were conducted by evaluating SYPRO orange dye fluorescence as a reporter for protein unfolding. Each reaction was conducted in a total volume of 100 μl comprising of 25mM HEPES (pH 7.5), 100 mM NaCl, 5–10 μM of protein and SYPRO orange dye (SIGMA) at a final dilution of 1:8000. These assays were performed in Synergy H4 microplate Reader at temperatures ranging from 25°C–65°C with a step size of 2°C, or by using a StepOnePlus RT-PCR machine employing a thermal ramp between 25–95°C (0.3°C per data point) for small molecule analysis, where compounds were evaluated at 10 μM (4% final DMSO concentration).Fluorescence was measured at each step with excitation at 470nm and emission at 570nm. For Aurora A, due to high background fluorescence, Sypro orange could not be used, so thermal melts were carried out and absorbance measurements were taken at 600nm, as described previously [75]. Due to different assays being used, the Tm values are not necessarily comparable for the two proteins. For all experiments, Tm values were calculated by fitting data to a Boltzmann sigmoidal curve after manual inspection of raw data for processing.

### Substrate phosphorylation of full length EphA3

HEK293T cells were cultured in Dulbecco’s Modified Eagle Medium (DMEM) containing 10% FBS, cells were transfected with EphA3 (WT or mutants) constructs as indicated. Full length EphA3 in pIRES2-EGFP vector was a kind gift from Dr. E. Pasquale (Sanford-Burnham Medical Research Institute, La Jolla, CA). Transfection was performed using a Ca_3_(PO_4_)_2_transfection method. In brief, for a 6 cm plate, 5 μg plasmid was suspended in 250 μl of 250 mM CaCl_2_ and incubated for 15 min, followed by the addition of equal volume of 2X BBS (BES (N,N-bis[2-hydroxyethyl]-2- aminoethanesulfonic acid)–buffered saline), mixed gently and incubated for a further 15–20 min. DNA complexes were directly added to the cells by gently swirling the plates. Cells were grown for 24 h and starved in DMEM without FBS for the next 18 h. Cells were then stimulated with 1 μg/ml Ephrin-A5-Fc or preclustered Ephrin-A5-Fc (Sigma, St. Louis, MO) for 10 minutes and washed with PBS and lysed in lysis buffer containing 50 mM Tris-HCl (pH 7.6), 150 mM NaCl, 10% glycerol, 1% Triton X-100, 1 mM EDTA and a protease inhibitor cocktail (EMD Millipore, Temecula, CA), as described in previous studies [36,76]. In brief, WT or mutant EphA3 in the total cell lysates (~1 ml) were immunoprecipitated using 2.5 μg anti-EphA3 antibody at 4°C for 3 h. 30 μl slurry of protein A/G plus agarose (Pierce, Rockford, IL) beads was added and incubated at 4°C overnight. Beads were washed with 2 x lysis buffer, 2x HNTG buffer (20 mM Hepes (pH 7.5), 150 mM NaCl 10% glycerol and 0.1% Triton X-100) and 2x kinase buffer (10 mM Hepes (pH 7.5), 25 mM MgCl_2_ and 10 mM MnCl_2_). Proteins bound on the beads were incubated with 10 μg of acid denatured rabbit muscle enolase (Sigma, St. Louis, MO) and 50 μM ATP in kinase buffer for 30 min at 25°C. SDS-PAGE sample buffer was used to stop the kinase reaction. Proteins were resolved on SDS-PAGE, transferred to PVDF membrane and phosphorylation of enolase and autophosphorylation of EphA3 was detected by immunoblotting with anti-p-tyr and anti-Y779 antibodies. Total EphA3 was analyzed by immunoblotting with anti-EphA3 antibody.

## Supporting Information

S1 Method Details describing the *in vitro* PK/LDH peptide phosphorylation assay, the microfluidic direct peptide phosphorylation assays, and peptide synthesis methodology. (DOCX)

S1 Table ATP Km values for the extended R-spine network mutants with and without the addition of STK-histidine. A residue equivalent to a drug resistance mutation occurring in Abl was also examined in EphA3 and is highlighted in red.
(DOCX)

S2 Table ATP Km values for additional mutants predicted to increase cavity size. (DOCX)

S1 Fig Full-length contrast hierarchical alignment showing evolutionary sequence constraints imposed on tyrosine kinase sequences. An alignment of representative human PTKs from diverse PTK sub-families is shown as a display alignment. The foreground set of PTK sequences (16807 sequences) and the background set of STK sequences (245319 sequences) are shown indirectly via consensus patterns and by column-wise amino acid frequencies (indicated by integer tenths) observed in the entire foreground versus background alignments. For example, a ‘5’ indicates that the corresponding amino acid occurs in 50–60% of the given (weighted) sequence set. Amino acid frequencies (denoted wt_res_freq) were determined from weighted sequences to account for overrepresented kinase families and evolutionary clades in the sequence data sets, and the number in parentheses indicates the number of sequences after down-weighting for redundancy. The alignment columns that were used to partition the foreground from the background sequences by the mcBPPS procedure are marked with black dots above the display alignment, and the degree to which the foreground amino acid distribution diverges from the background amino acid distribution at each position is plotted as a red histogram. Each PTK sequence in the display alignment is numbered corresponding to the Uniprot sequence corresponding to the Uniprot ID given next to the PTK name. The STK-histidine that is selectively lost in PTKs is highlighted in the alignment with a red rectangle.
(TIFF)

S2 Fig Structural locations of all PTK-conserved residues. All PTK conserved residues identified in S1 Fig are shown mapped to human EphA3 crystal structure (PDB: 3fy2) and annotated based on structural location. Most PTK-conserved residues form structural interaction networks, as highlighted in the structure.
(TIFF)

S3 Fig Conservation of extended R-spine network residues in PTK, TKL and STKs across various evolutionary phyla. The residues are numbered according to EphA3 numbering. The PTK-conserved residues are not conserved in STKs for any of the phyla studied. The TKLs show partial conservation of PTK-conserved residues in eukaryotes closer to Metazoans. Amoebozoa have sequences in both PTK and TKL families with PTK-conserved residues. For comparison to Metazoans, two phyla not part of Unikonts are shown that do not have PTK-conserved residues in TKL families.
(TIFF)

S4 Fig Comparisons of interaction energies associated with extended R-spine residues and R-spine-Asp in PTK and STK crystal structures (pairwise residue energy calculation is described in methods). Data is represented as heatmaps with the scale of interactions indicated in the figure. The residues are numbered according to EphA3 structure (pdb id 3fy2). **A-B)** Extended R-spine residues (y-axis) and their interactions with surrounding residues in STKs and PTKs respectively. Position 806 corresponds to R-spine-Asp and position 738 corresponds to STK-histidine. **C)** Interaction energies of extended R-spine residues and STK-histidine (or equivalent residues) with R-spine-Asp for major classes of protein kinases. Note the absence of interaction energy between STK-histidine (S738 position) and R-spine-Asp in PTKs and the absence of F871 and R-spine-Asp interaction in STKs. Y810 is not shown here because it interacts with R-spine-Asp only through alpha helix backbone hydrogen bonds.
(TIFF)

S5 Fig Conservation of amino acids and secondary structure in the I-helix. The upper panels show the DSSP derived secondary structure annotation in all PTKs, STKs and TKLs studied in this paper. ‘L’ stands for loop, ‘H’ stands for helix ‘S’ stands for sheet and ‘G’ & ‘T’ stand for turns. The position of F871 is shown in the PTK panel and the position of a proline conserved in STKs that causes a kink in the I-helix is also shown. The bottom panel is a heatmap showing the conservation of the 20 amino acids in the 7 major groups of kinases. As can be seen from the heatmap, a proline is mostly conserved in all STK groups, but a leucine is present in TKLs and PTKs leading to a longer I-helix.
(TIFF)

S6 Fig Side chain flexibility of R-spine-Asp in EphA3 WT and mutants in selected PTKs and STKs. In each of these panels, chi1 and chi2 dihedral angles (as defined in Gromacs) are plotted for the R-spine-Asp. For clarity, the chi1-chi2 angles in PTK simulations are shown in green and for STKs are shown in blue. **A)** EphA3 mutant and WT side-chain flexibility is shown for extended R-spine mutants. Also shown in the bottom panel are the plots showing reduction in fluctuations in R-spine-Asp when STK-histidine (S738H) is introduced in WT and F871A background. In both cases, the side-chain fluctuations are damped. Note that the TKL-like state in EphA3 (last panel, S738H mutant) resembles the reduced fluctuations seen in all STKs (see part B). **B)** Side chain flexibility of R-spine-Asp in selected PTKs and STKs. Note that all STKs show similar chi1-chi2 plots, but different PTKs show varying degree of flexibility of the R-spine-Asp. Such variation could arise due to family-specific variations in the kinase core.
(TIFF)

S7 Fig Triple mutant analysis of EphA3. All three extended R-spine network residues in EphA3 are replaced by those observed in AuroraA (F871L+M734L+Y810L) andchi1 and chi2 dihedral angles of the R-spine-Asp are shown for the triple mutant in the presence and absence of the STK-histidine.
(TIFF)

S8 Fig Stability analysis of extended R-spine mutants using clustering analysis (see Materials and Methods section and Fig 3 for details). The number of clusters reflects the stability of R-spine, ATP-binding pocket and extended R-spine residues. Deletion of F871A leads to the largest increase in fluctuations in the R-spine. Compared to WT, all three mutants show destabilization and increased fluctuations of the R-spine and ATP binding pocket.
(TIFF)

S9 Fig Tm measurements of EphA3 mutants and AuroraA mutants. The Tm measurements were calculated from triplicate runs. In each of these measurements, temperature was increased with a step size of two degree Celsius giving a ramp rate of ~1 degree/min. Aurora A stability measurements were done using O.D. 600nm as a measure of aggregation. EphA3 stability was monitored by dye binding assay using Sypro Orange dye with excitation 470nm and emission 570nm. The normalized fluorescence values are shown for each mutant.
(TIFF)

S10 Fig Graphs showing the fits to the data that were used to calculate Km values for ATP. The experiments were done with an entire series of ATP concentrations replicated at least 5 times for each mutant.
(TIFF)

S11 Fig Specific activity of Aurora A mutants. Addition of the extended R-spine network residues (L374F and L244M) to a mutant lacking the STK-histidine (H248) leads to a partial rescue of activity.
(TIFF)

S12 Fig Thermal stability of R-spine mutants and IC_50_determination for a type I (dasatinib) and type II (ponatinib) EphA3 inhibitor. **A)** Tm values determined for indicated EphA3 proteins in DSF inhibitor assay conditions (4% v/v DMSO). Results corroborate thermal stabilities of EphA3 and mutants observed in initial thermal melt experiments presented in Fig 4c. Data shown are from two replicates, ± SD. **B-C)** IC_50_ value determination for dasatinib (B) or ponatinib (C) comparing WT and F781A mutant in a direct peptide-based kinase assay. IC_50_ values for both inhibitors are essentially identical for both WT and F871A EphA3.
(TIFF)
