# Correction: Hydrophobic Core Variations Provide a Structural Framework for Tyrosine Kinase Evolution and Functional Specialization

**DOI:** 10.1371/journal.pgen.1006265

**Published:** 2016-08-11

**Authors:** Smita Mohanty, Krishnadev Oruganty, Annie Kwon, Dominic P. Byrne, Samantha Ferries, Zheng Ruan, Laura E. Hanold, Samiksha Katiyar, Eileen J. Kennedy, Patrick A. Eyers, Natarajan Kannan

There is an error in reference 52. The correct reference is: Bosc N, Wroblowski B, Aci-Sèche S, Meyer C, Bonnet P (2015) A Proteometric Analysis of Human Kinome: Insight into Discriminant Conformation-Dependent Residues. ACS Chem Biol 10: 2827–2840. doi: 10.1021/acschembio.5b00555. pmid: 26411811
