# Supplementary material for: Hydrophobic Core Variations Provide a Structural Framework for Tyrosine Kinase Evolution and Functional Specialization
Source: PLoS Genet. 2016 Feb 29;12(2):e1005885. doi: 10.1371/journal.pgen.1005885 (PMC4771162; doi:10.1371/journal.pgen.1005885)
Supplement: S1 Method — (DOCX) [file pgen.1005885.s001.docx]

**Supplementary method**

We employed a PK/LDH coupled enzyme assay in 96 well format for enzyme kinetics, or a direct peptide phosphorylation assay using a Perkin Elmer EZ Reader in 384 well format. In the first assay, reaction progress (conversion of ATP to ADP) was monitored by a coupled reaction that leads to oxidation of NADH. The NADH and ATP reagents used and the enzymes PK/LDH were purchased from Sigma. For each of the proteins including the wild type and the mutants of EphA3, Km for ATP and peptide were determined. These reactions were carried out at 35°C in a total reaction volume of 50 µl and reduction in absorbance was measured at 340nm every 2 minutes using absorbance mode of Synergy H4 microplate Reader. The reaction mixture comprised of 100 mM Tris (pH 8.1), 300 mM NaCl, 20 mM MgCl_2_, 1 mM PEP, 500 µM NADH, 400 µM peptide substrate (NH2-KQWDNYEFIW-COOH [67], obtained from Biomatik corporation) and 100-200 nM of EphA3 (depending on the mutant protein). Each of the reactions was initiated by addition of ATP at varying concentrations ranging from 2.5 mM to 25 µM. Before performing the assays, the EphA3 proteins were incubated with 5 mM ATP and 10 mM MgCl_2_ at room temperature for two hours to allow for complete autophosphorylation of EphA3, which is a prerequisite for activity. Subsequently, excess ATP was removed by repeated cycles of concentrating the protein and dilution with dialysis buffer (25mM HEPES at pH 7.5 and 500mM NaCl). While conducting the assay, a no-peptide control is also included to account for any ATPase activity or auto-phosphorylation exhibited by the protein. The amount of protein used was optimized such that the kinase reaction was at least 25 times slower than the coupled PK/LDH reaction. This was achieved by carrying out the assay at varying concentrations and choosing an enzyme concentration within the linear range of activity vs protein concentration. Typically the final concentration of enzyme used in this assay ranged from 200-500 nM, depending on the mutant protein. Since all the reactions were carried out in excess of peptide or ATP, the two substrate kinase reaction was simplified into a single substrate reaction and Km and Vmax were determined by fitting the data to the Michaelis-Menton equation (Graphpad PRSIM). Specific activity for the proteins was estimated by taking a ratio of the Vmax and the concentration of enzymes used in the assay. For peptide Km, the ATP concentration used was 1 mM for all mutants with the peptide concentration varied from 40 µM to 900 µM. Optimization of pH and addition of 5% DMSO was required for peptide Km since published Km values could only be obtained at a pH of 8.1. The peptide Km of WT EphA3 increases upon lowering or raising the pH from 8.1. Similar assay conditions with an Aurora peptide (NleRRRLSFFFWAKK)) [69] were employed to determine the specific activity for Aurora proteins. The peptide concentration was kept at 200 μM and ATP concentration was 2 mM for Aurora specific activity assays. In microfluidic EphA3 assays, 100 ng of protein was evaluated in a kinetic experiment with 5 μM EphA3 fluorescent peptide substrate (5-FAM-EFPIYDFLPAKKK-CONH_2_). Phosphorylation was limited to <20% in the presence of 0.2 mM ATP, 5 mM MgCl_2_, and inhibitor (final DMSO concentration 1% (v/v)). Peptide phosphorylation was calculated directly from differentiation of the phosphopeptide:peptide ratio using EZ Reader software, and IC_50_ values were determined using GraphPad software.

The AuroraA substrate peptide was prepared using standard Fmoc-based solid-phase peptide synthesis on rink amide MBHA resin (50 µmol). Deprotections were performed in a solution of 25% piperide in NMP for 25 minutes followed by 3 washes with NMP for 30 seconds each. Amino acid couplings were performed using a 0.5 M solution of Fmoc-protected amino acid (1 mL, 0.5 mmol, 10 equiv.), 0.5 M HCTU (0.99 mL, 0.495 mmol, 9.9 equiv.) and diisopropylethylamine (174 µL, 1 mmol, 20 equiv.). The reaction mixture was allowed to bubble for 45 minutes before washing three times with NMP. After deprotecting the final amino acid, the peptide was cleaved from the resin in a solution containing 95% trifluoroacetic acid, 2.5% triisopropylsilane, and 2.5% water for 4 hours. The peptide was then filtered through glass wool into methyl *t*-butyl ether, and the resulting precipitate was collected by centrifugation at 4°C. After decanting the supernatant and air-drying the pellet, the crude peptide was dissolved in methanol for characterization and purification by LC/MS and reverse-phase HPLC using a Zorbax SB-C18 5 µm column and an Agilent 1200 series HPLC system coupled to the Agilent 1620 LC/MS. Purification was performed using a flow rate of 4 mL/min and a gradient of 10%-100% acetonitrile containing 0.1% trifluoroacetic acid. The peptide was quantified using the extinction coefficient of 6590 M^-1^ cm^-1^ for tryptophan in 10 mM Tris pH 8. Peptide molecular weight = 1753.4 (expected = 1754.1).
