# Supplementary material for: Hydrophobic Core Variations Provide a Structural Framework for Tyrosine Kinase Evolution and Functional Specialization
Source: PLoS Genet. 2016 Feb 29;12(2):e1005885. doi: 10.1371/journal.pgen.1005885 (PMC4771162; doi:10.1371/journal.pgen.1005885)
Supplement: S1 Table — A residue equivalent to a drug resistance mutation occurring in Abl was also examined in EphA3 and is highlighted in red. (DOCX) [file pgen.1005885.s002.docx]

| Protein | Description | ATP Km (µM) | Peptide Km  (µM) | Specific activity (nano moles min^-1^ µmoles^-1^ ) |
| --- | --- | --- | --- | --- |
| EphA3 WT |  | 81 (± 11) | 50.3 ± 7 | 4.72 (±0.11) |
| D806N | R-spine Asp | - | - | Not detected |
| R789K | Substrate pocket | - | - | Not detected |
| W790Y | Substrate pocket | - | - | Not detected |
| F871A | Extended R-spine | 179 (± 22) | 58 ± 14 | 2.19 (±0.12) |
| M734L | Extended R-spine | 263 (± 62) | 72 ± 10 | 1.33 (±0.12) |
| Y810L | Extended R-spine | 229 (± 16) | 91 ± 20 | 0.89 (±0.06) |
| S738H | TKL-like state with STK- histidine | 150 (± 8) | 57 ± 9 | 4.17 (±0.29) |
| F871A+S738H | Extended R-spine rescue with STK-histidine | 130 (± 14) | 62 ± 5 | 4.73 (±0.02) |
| M734L+S738H | Extended R-spine rescue with STK-histidine | 160 (± 5) | 68 ± 6 | 6.56 (±0.17) |
| Y810L+S738H | Extended R-spine rescue with STK-histidine | 162 (± 46) | 94 ± 22 | 1.11 (±0.06) |
| M734T | Extended R-spine mutation implicated in drug resistance | 423 (± 52) | - | 1.56 (±0.1) |
| M734F | Cavity filling mutation found in some AGC kinases | 456 ± 39 | - | - |
