# Supplementary material for: Hydrophobic Core Variations Provide a Structural Framework for Tyrosine Kinase Evolution and Functional Specialization
Source: PLoS Genet. 2016 Feb 29;12(2):e1005885. doi: 10.1371/journal.pgen.1005885 (PMC4771162; doi:10.1371/journal.pgen.1005885)
Supplement: S2 Table — (DOCX) [file pgen.1005885.s003.docx]

| Protein | ATP Km (µM) |
| --- | --- |
| M734V | 306 ± 21 |
| M734V+S738H | 167 ± 19 |
| Y810V | 490 ± 218 |
| Y810V+S738H | 213 ± 4 |
