# Supplementary figures and images for: Hydrophobic Core Variations Provide a Structural Framework for Tyrosine Kinase Evolution and Functional Specialization
Source: PLoS Genet. 2016 Feb 29;12(2):e1005885. doi: 10.1371/journal.pgen.1005885 (PMC4771162; doi:10.1371/journal.pgen.1005885)

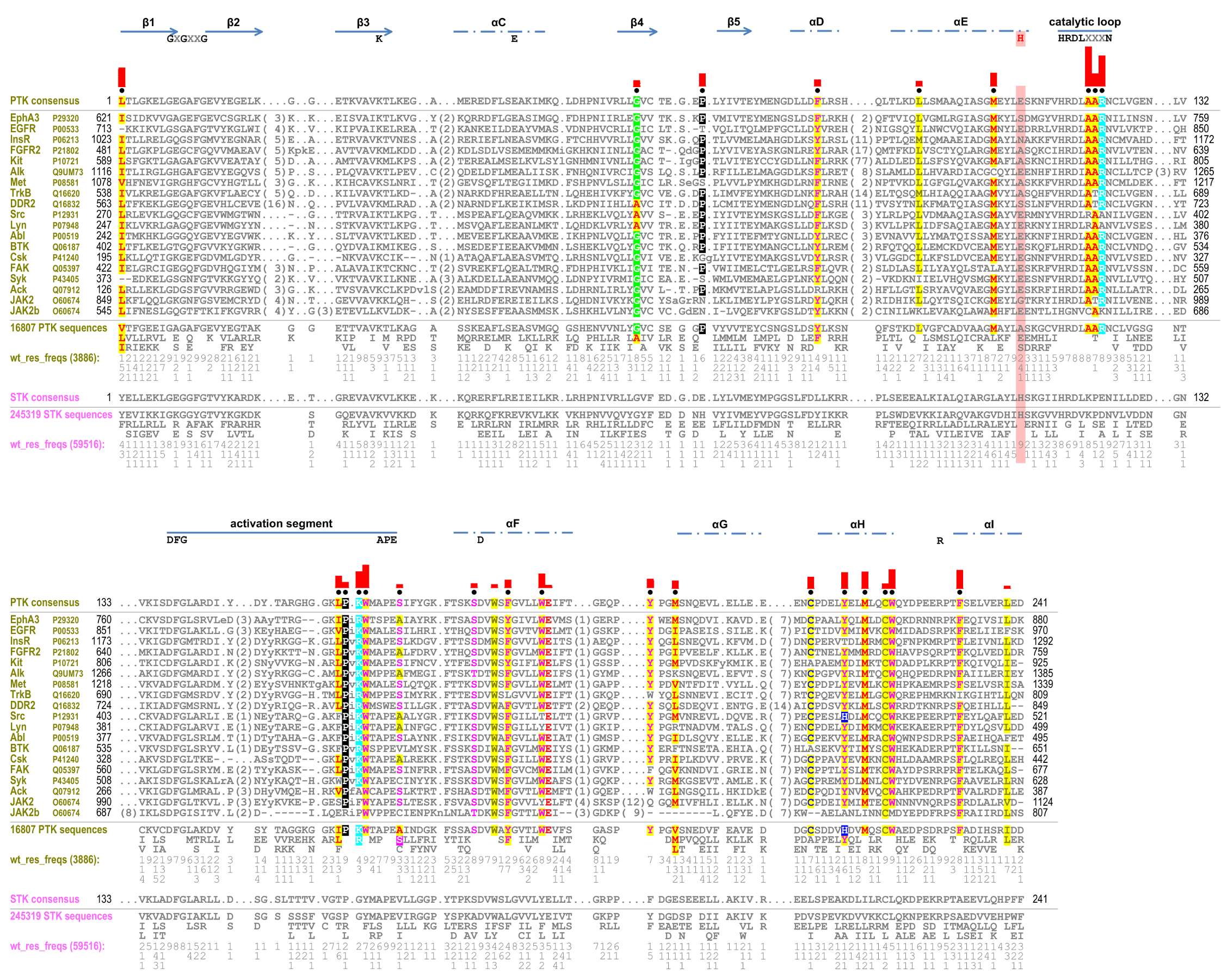

Supplement: S1 Fig — An alignment of representative human PTKs from diverse PTK sub-families is shown as a display alignment. The foreground set of PTK sequences (16807 sequences) and the background set of STK sequences (245319 sequences) are shown indirectly via consensus patterns and by column-wise amino acid frequencies (indicated by integer tenths) observed in the entire foreground versus background alignments. For example, a ‘5’ indicates that the corresponding amino acid occurs in 50–60% of the given (weighted) sequence set. Amino acid frequencies (denoted wt_res_freq) were determined from weighted sequences to account for overrepresented kinase families and evolutionary clades in the sequence data sets, and the number in parentheses indicates the number of sequences after down-weighting for redundancy. The alignment columns that were used to partition the foreground from the background sequences by the mcBPPS procedure are marked with black dots above the display alignment, and the degree to which the foreground amino acid distribution diverges from the background amino acid distribution at each position is plotted as a red histogram. Each PTK sequence in the display alignment is numbered corresponding to the Uniprot sequence corresponding to the Uniprot ID given next to the PTK name. The STK-histidine that is selectively lost in PTKs is highlighted in the alignment with a red rectangle. (TIFF) [file pgen.1005885.s004.tiff]

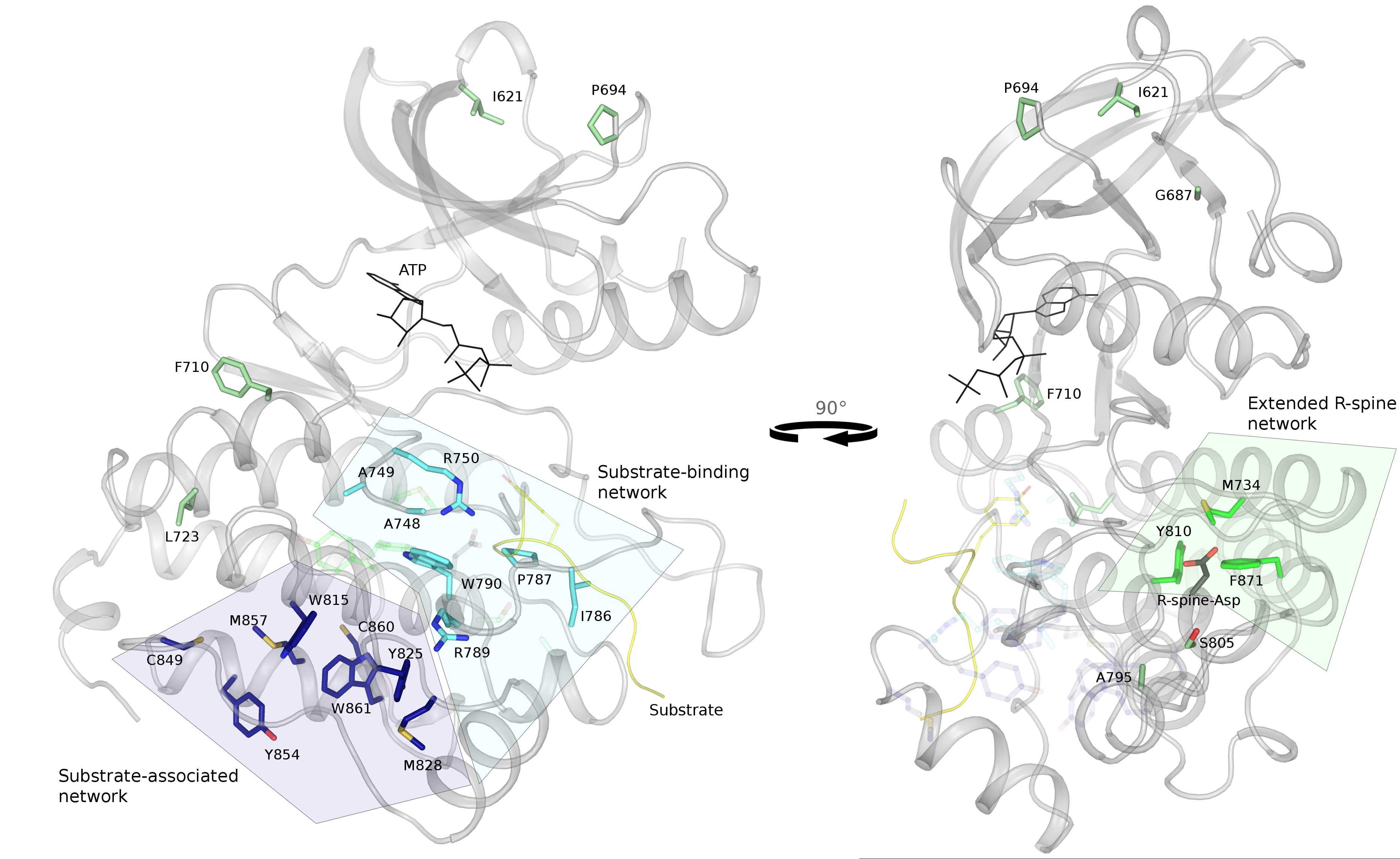

Supplement: S2 Fig — All PTK conserved residues identified in S1 Fig are shown mapped to human EphA3 crystal structure (PDB: 3fy2) and annotated based on structural location. Most PTK-conserved residues form structural interaction networks, as highlighted in the structure. (TIFF) [file pgen.1005885.s005.tiff]

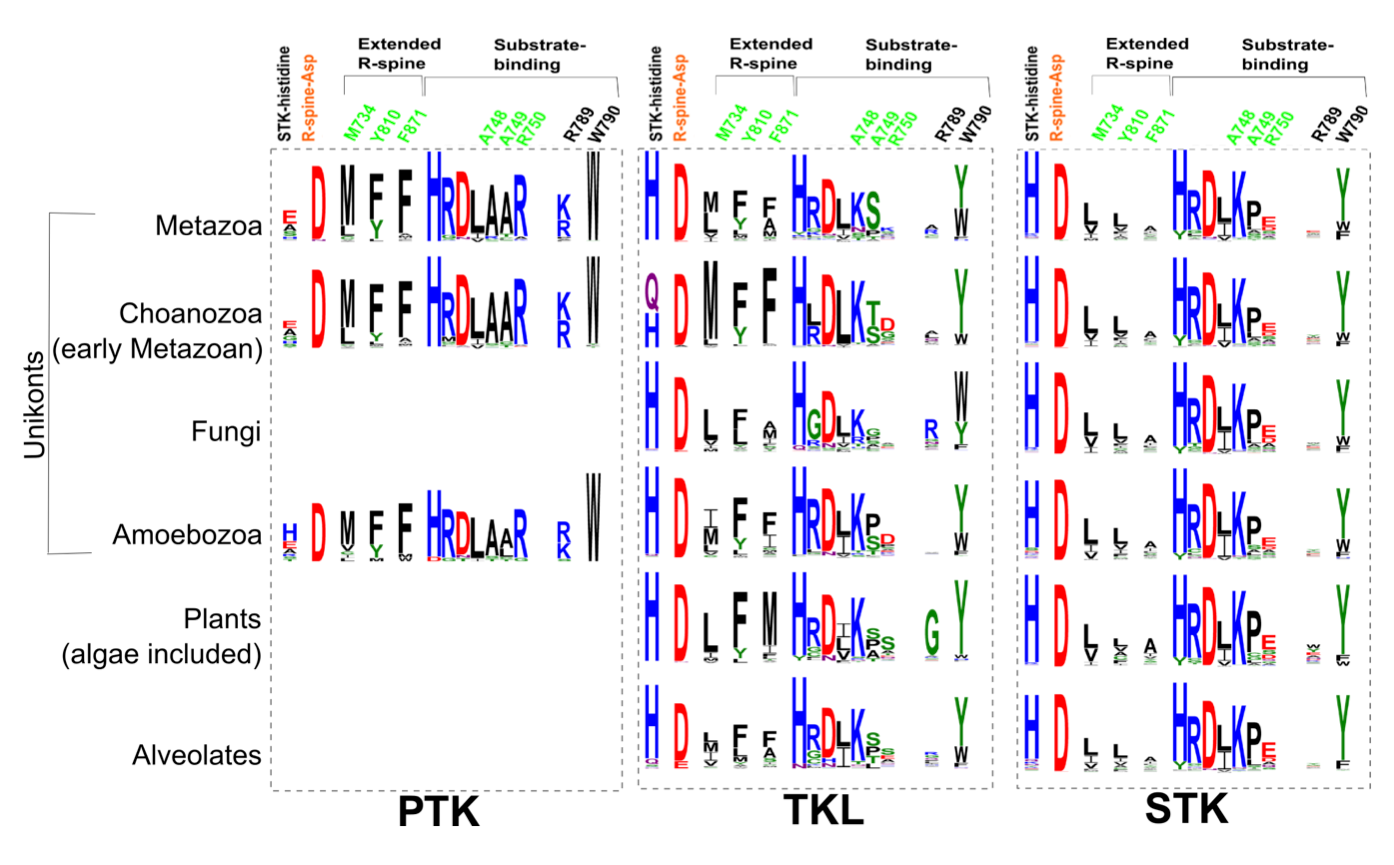

Supplement: S3 Fig — The residues are numbered according to EphA3 numbering. The PTK-conserved residues are not conserved in STKs for any of the phyla studied. The TKLs show partial conservation of PTK-conserved residues in eukaryotes closer to Metazoans. Amoebozoa have sequences in both PTK and TKL families with PTK-conserved residues. For comparison to Metazoans, two phyla not part of Unikonts are shown that do not have PTK-conserved residues in TKL families. (TIFF) [file pgen.1005885.s006.tiff]

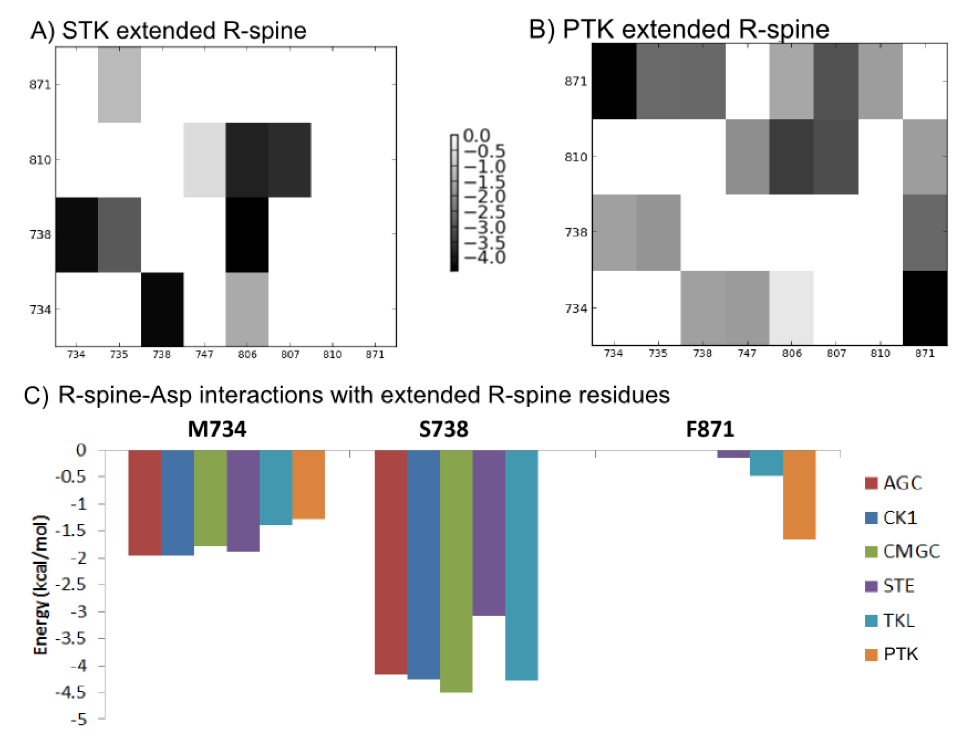

Supplement: S4 Fig — Data is represented as heatmaps with the scale of interactions indicated in the figure. The residues are numbered according to EphA3 structure (pdb id 3fy2). A-B) Extended R-spine residues (y-axis) and their interactions with surrounding residues in STKs and PTKs respectively. Position 806 corresponds to R-spine-Asp and position 738 corresponds to STK-histidine. C) Interaction energies of extended R-spine residues and STK-histidine (or equivalent residues) with R-spine-Asp for major classes of protein kinases. Note the absence of interaction energy between STK-histidine (S738 position) and R-spine-Asp in PTKs and the absence of F871 and R-spine-Asp interaction in STKs. Y810 is not shown here because it interacts with R-spine-Asp only through alpha helix backbone hydrogen bonds. (TIFF) [file pgen.1005885.s007.tiff]

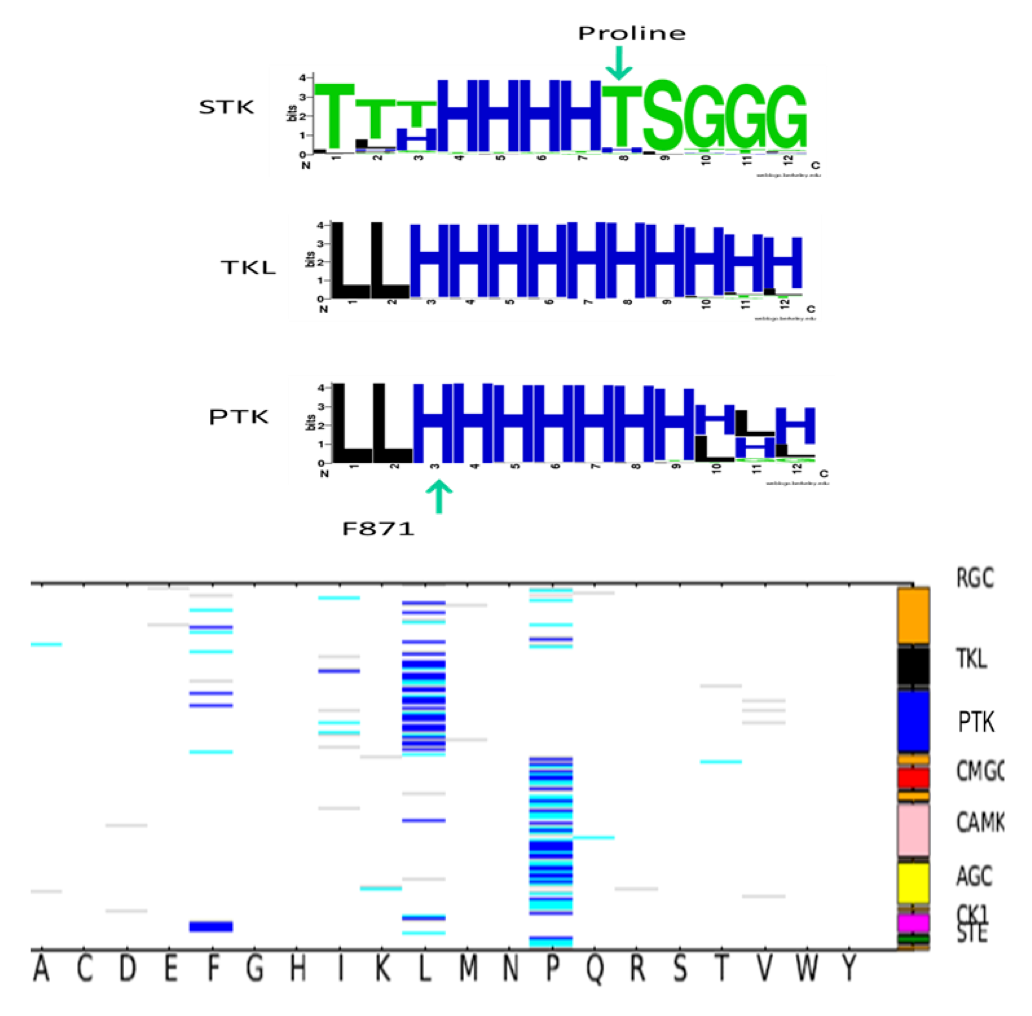

Supplement: S5 Fig — The upper panels show the DSSP derived secondary structure annotation in all PTKs, STKs and TKLs studied in this paper. ‘L’ stands for loop, ‘H’ stands for helix ‘S’ stands for sheet and ‘G’ & ‘T’ stand for turns. The position of F871 is shown in the PTK panel and the position of a proline conserved in STKs that causes a kink in the I-helix is also shown. The bottom panel is a heatmap showing the conservation of the 20 amino acids in the 7 major groups of kinases. As can be seen from the heatmap, a proline is mostly conserved in all STK groups, but a leucine is present in TKLs and PTKs leading to a longer I-helix. (TIFF) [file pgen.1005885.s008.tiff]

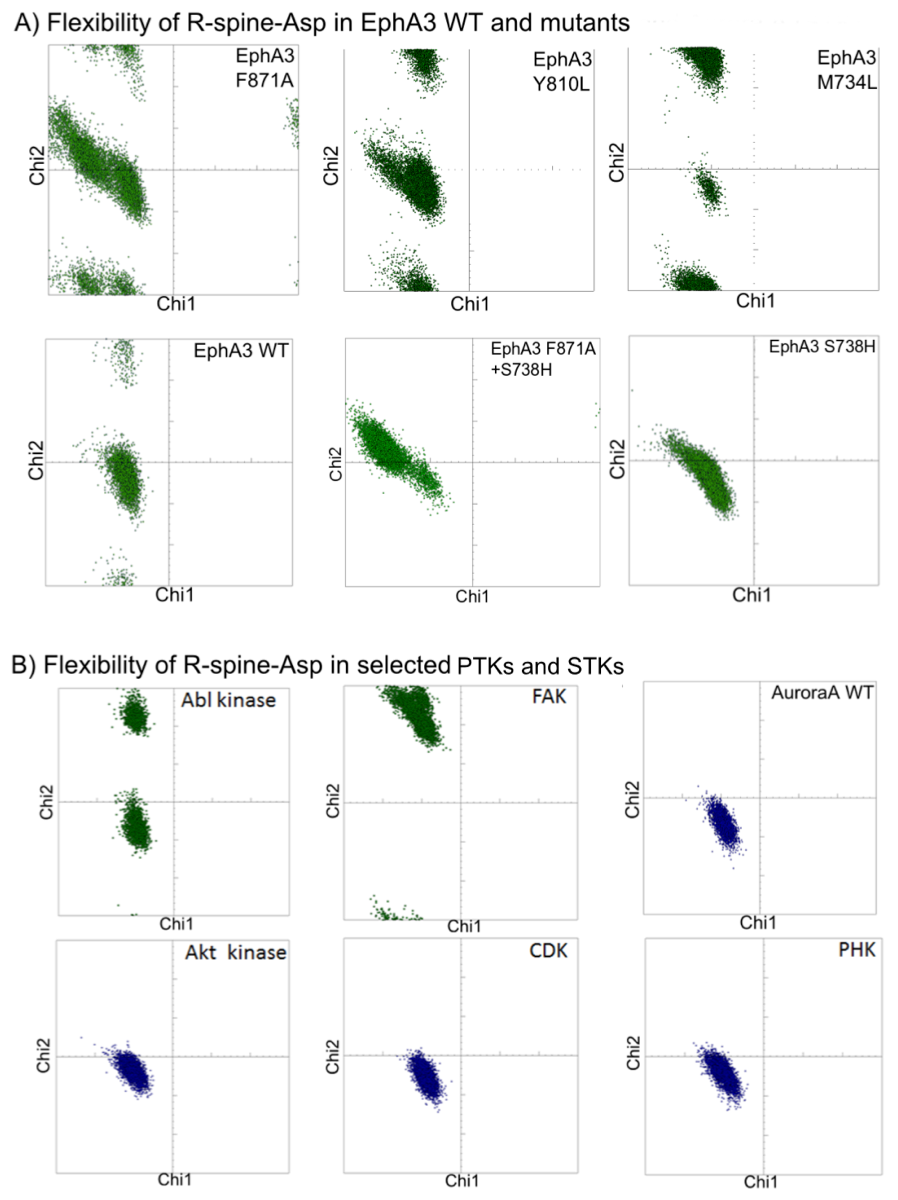

Supplement: S6 Fig — In each of these panels, chi1 and chi2 dihedral angles (as defined in Gromacs) are plotted for the R-spine-Asp. For clarity, the chi1-chi2 angles in PTK simulations are shown in green and for STKs are shown in blue. A) EphA3 mutant and WT side-chain flexibility is shown for extended R-spine mutants. Also shown in the bottom panel are the plots showing reduction in fluctuations in R-spine-Asp when STK-histidine (S738H) is introduced in WT and F871A background. In both cases, the side-chain fluctuations are damped. Note that the TKL-like state in EphA3 (last panel, S738H mutant) resembles the reduced fluctuations seen in all STKs (see part B). B) Side chain flexibility of R-spine-Asp in selected PTKs and STKs. Note that all STKs show similar chi1-chi2 plots, but different PTKs show varying degree of flexibility of the R-spine-Asp. Such variation could arise due to family-specific variations in the kinase core. (TIFF) [file pgen.1005885.s009.tiff]

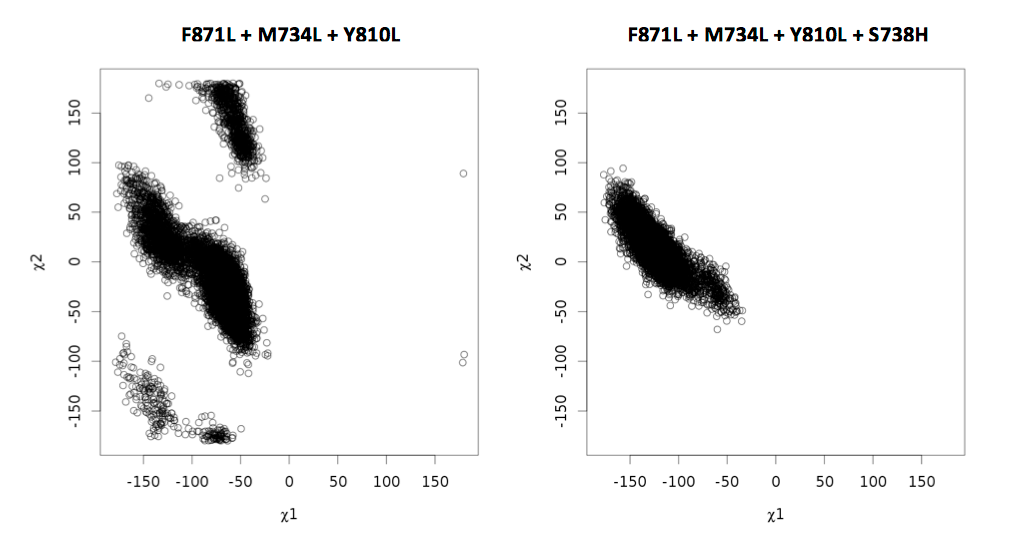

Supplement: S7 Fig — All three extended R-spine network residues in EphA3 are replaced by those observed in AuroraA (F871L+M734L+Y810L) andchi1 and chi2 dihedral angles of the R-spine-Asp are shown for the triple mutant in the presence and absence of the STK-histidine. (TIFF) [file pgen.1005885.s010.tiff]

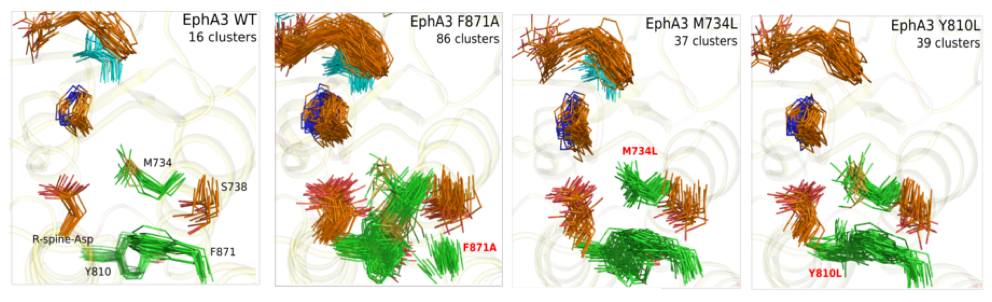

Supplement: S8 Fig — The number of clusters reflects the stability of R-spine, ATP-binding pocket and extended R-spine residues. Deletion of F871A leads to the largest increase in fluctuations in the R-spine. Compared to WT, all three mutants show destabilization and increased fluctuations of the R-spine and ATP binding pocket. (TIFF) [file pgen.1005885.s011.tiff]

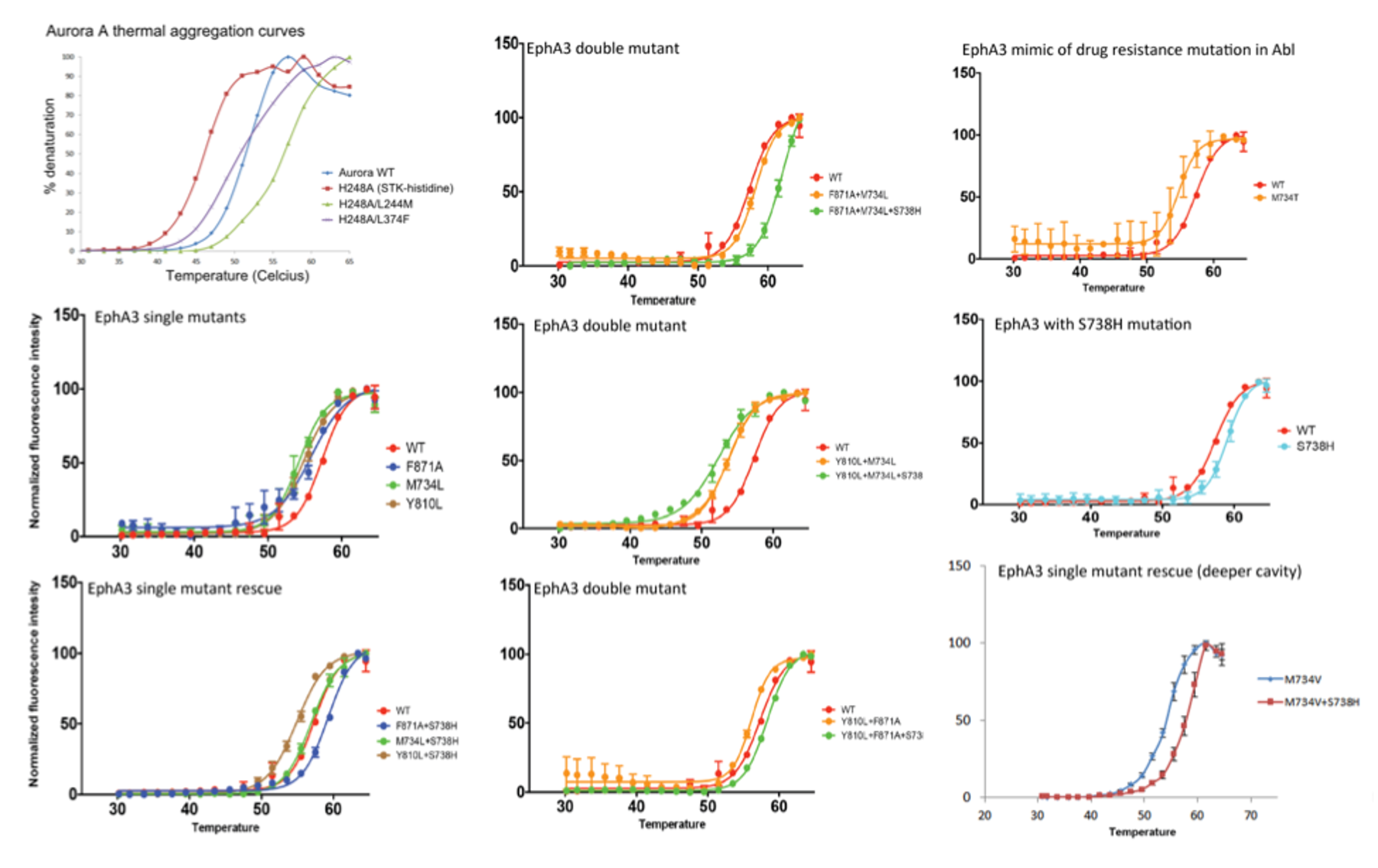

Supplement: S9 Fig — The Tm measurements were calculated from triplicate runs. In each of these measurements, temperature was increased with a step size of two degree Celsius giving a ramp rate of ~1 degree/min. Aurora A stability measurements were done using O.D. 600nm as a measure of aggregation. EphA3 stability was monitored by dye binding assay using Sypro Orange dye with excitation 470nm and emission 570nm. The normalized fluorescence values are shown for each mutant. (TIFF) [file pgen.1005885.s012.tiff]

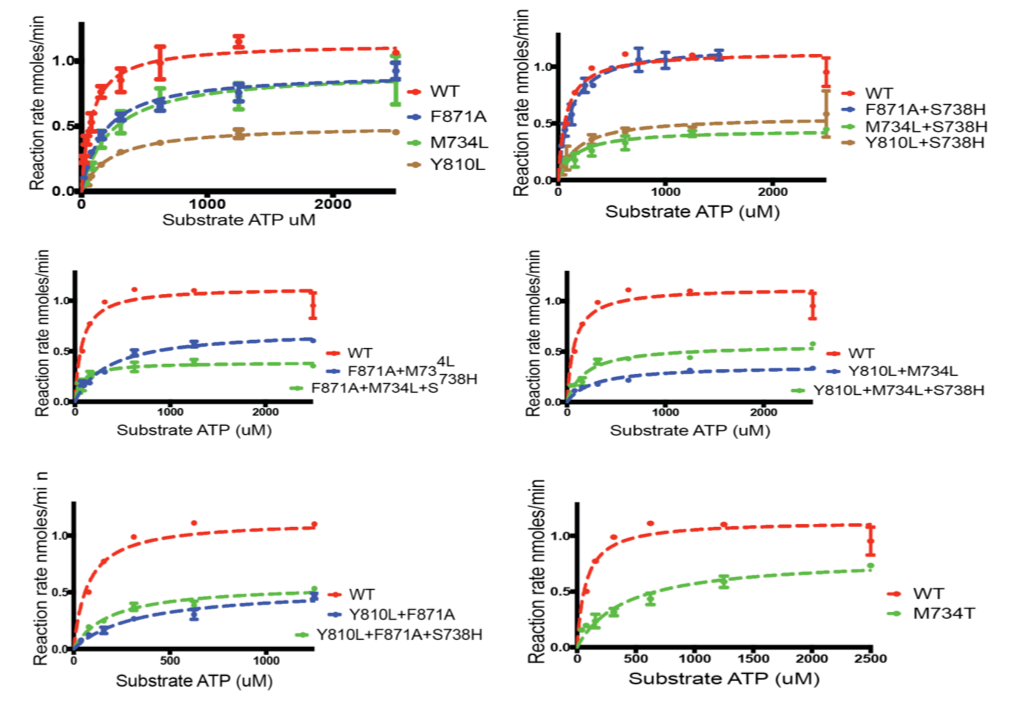

Supplement: S10 Fig — The experiments were done with an entire series of ATP concentrations replicated at least 5 times for each mutant. (TIFF) [file pgen.1005885.s013.tiff]

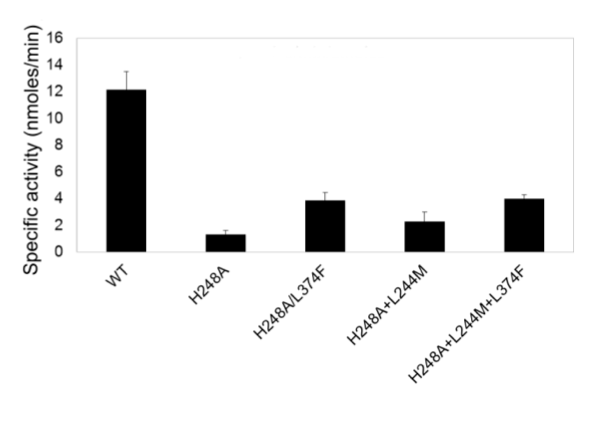

Supplement: S11 Fig — Addition of the extended R-spine network residues (L374F and L244M) to a mutant lacking the STK-histidine (H248) leads to a partial rescue of activity. (TIFF) [file pgen.1005885.s014.tiff]

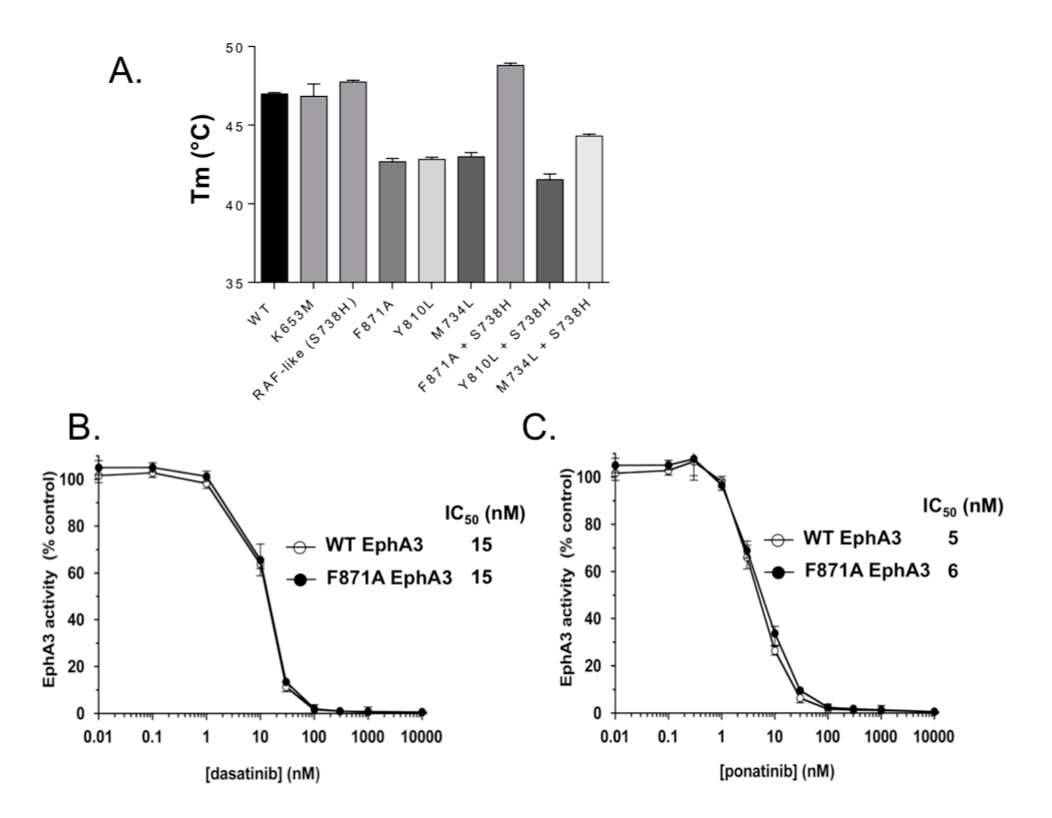

Supplement: S12 Fig — A) Tm values determined for indicated EphA3 proteins in DSF inhibitor assay conditions (4% v/v DMSO). Results corroborate thermal stabilities of EphA3 and mutants observed in initial thermal melt experiments presented in Fig 4c. Data shown are from two replicates, ± SD. B-C) IC50 value determination for dasatinib (B) or ponatinib (C) comparing WT and F781A mutant in a direct peptide-based kinase assay. IC50 values for both inhibitors are essentially identical for both WT and F871A EphA3. (TIFF) [file pgen.1005885.s015.tiff]
